# Identifying hotspots of invasive alien terrestrial vertebrates in Europe to assist transboundary prevention and control

**DOI:** 10.1038/s41598-020-68387-3

**Published:** 2020-07-15

**Authors:** Ester Polaina, Tomas Pärt, Mariano R. Recio

**Affiliations:** 1 Department of Ecology, Swedish University of Agricultural Sciences, Box 7044, 75007 Uppsala, Sweden; 2 Departamento de Biología y Geología, Física y Química Inorgánica, Universidad Rey Juan Carlos, ESCET, Tulipán s/n, 28933 Móstoles, Madrid Spain

**Keywords:** Biogeography, Conservation biology, Ecological modelling, Invasive species

## Abstract

This study aims to identify environmentally suitable areas for 15 of the most harmful invasive alien terrestrial vertebrates (IATV) in Europe in a transparent and replicable way. We used species distribution models and publicly-available data from GBIF to predict environmental suitability and to identify hotspots of IATV accounting for knowledge gaps in their distributions. To deal with the ecological particularities of invasive species, we followed a hierarchical approach to estimate the global climatic suitability for each species and incorporated this information into refined environmental suitability models within Europe. Combined predictions on environmental suitability identified potential areas of IATV concentrations or hotspots. Uncertainty of predictions identified regions requiring further survey efforts for species detection. Around 14% of Europe comprised potential hotspots of IATV richness, mainly located in northern France, UK, Belgium and the Netherlands. IATV coldspots covered ~ 9% of Europe, including southern Sweden and Finland, and northern Germany. Most of Europe (~ 77% area) comprised uncertain suitability predictions, likely caused by a lack of data. Priorities on prevention and control should focus on potential hotspots where harmful impacts might concentrate. Promoting the collection of presence data within data-deficient areas is encouraged as a core strategy against IATVs.

## Introduction

Invasive alien species (IAS) are the second greatest cause of global biodiversity loss and endangerment, after habitat destruction, and pose an increasing threat to human economies and native ecosystems^1,2^. The varied insidious impacts caused by IAS have prolifically been reported for island ecosystems due to the particular fragility of these environments^3^. However, alien species have invaded all kind of ecosystems and their impacts at large continental scales are of great concern for the conservation of natural and human systems^4^. In the United States alone, IAS are considered a threat for 42% of endangered species, and involve annual costs of U.S. $137 billion^5^. In Europe, IAS are responsible for estimated damage costs (i.e. excluding management costs) of €10–20 billion per year^6^. Approximately 14,000 alien species were reported in 2015 in Europe, of which ~ 10–15% are considered invasive^7^. However, the potential economic and ecological impact of about 90% of these species remains unknown^8^. Therefore, improving knowledge about invasion patterns, risks, and impacts is still required.

Control of IAS is included as a priority to halt biodiversity loss in global initiatives, such as the Convention on Biological Diversity^9^. Europe has specific legislation on IAS prevention and management; however, transboundary cooperation is necessary to guarantee their compliance and effectiveness, including that from bordering non-EU countries^10,11^. To reduce the expansion and associated deleterious impacts of IAS, it is of critical importance to identify and characterize priority areas of management that include hotspots of achieved or potential invasion of the most harmful IAS at a continental level. Research efforts in this direction can assist decision-making and inform policies that focus on cost-efficient management strategies^12^.

Anticipating areas where IAS are likely to persist can be achieved using species distribution models (SDM). These models identify the relationships between current species’ presence and the abiotic (usually climatic variables) and biotic (e.g. habitat, species interactions) factors that are associated with species survival and establishment in a given area^13^. However, IAS violate the theoretical assumptions of traditional SDM methods. Niche transferability does not always occur because invasive species can adapt to new conditions within invaded areas and, normally, they are not in equilibrium with the environment (i.e. their absence may not indicate unsuitable conditions but rather lack of detection, dispersal limitations or low propagule pressure^14^). To overcome these limitations, when real absences are missing, SDM frameworks tailored for invasive species incorporate both native and invasive distribution ranges to include all recorded climatic conditions where the species may persist. In hierarchical approaches, this information is included in a global model, which approximates the global climatic suitability for the species, to partially overcome niche transferability issues. These results are used to weigth the pseudo-absences at the regional level, providing a higher weight (i.e. closer to a real absence) to areas where global suitability is lower and, therefore, regional suitability is likely to be low (vs. non-occupied)^15^.

Among alien species, invasive alien terrestrial vertebrates (hereafter only IATV) abound and are of particular relevance in Europe due to their high establishment rates and the broad range of impacts they cause within vast areas^16,17^. IATV threaten native species by predation, competition, hybridization and spread of diseases, and have devastating impacts on socioeconomic systems and on public and animal health^18^. As reported by the DAISIE Project (“Delivering Alien Invasive Species Inventories for Europe”), there are around 270 IATV species in Europe, of which 15 are in the expert-based ranking list of the “100 of the Most Invasive Alien Species in Europe”, a representative sample of diverse harmful impacts known to occur in Europe^18^. These 15 harmful IATV comprise nine mammals, four birds, one amphibian and one reptile (Table 1). Their negative impacts include damage to crops, wood plantations and recreational areas by *Cervus nippon* or *Branta canadensis*^19,20^, transmission of diseases such as rabies or Lyme disease by *Procyon lotor* or *Tamias sibiricus*^21,22^, and introduction in Europe by *Lithobates catesbeianus* of the lethal chytrid fungus that threatens amphibian populations worldwide^23^.

**Table 1:** List of the 15 invasive alien terrestrial vertebrates (IATV) included in the DAISIE’s list of 100 of the worst alien species in Europe^67^.

Scientific name	Common names	Native range
**Mammals**		
*Cervus nippon* (Temminck, 1836)	Sika deer, Japanese deer	Japan, Taiwan, China, Far Eastern Russia
*Myocastor coypus* (Molina, 1782)	Coypu, nutria	Argentina, Bolivia, southern Brazil, Chile, Paraguay, Uruguay
*Neovison vison* (Schreber, 1777)	American mink	Canada and United States, except Arizona and the dry parts of California, Nevada, Utah, New Mexico, western Texas
*Nyctereutes procyonoides* (Gray, 1834)	Racoon dog, mangut, tanuki, neoguri	China, Japan, Macau, Mongolia, North and South Korea, Vietnam
*Ondatra zibethicus* L., 1766	Muskrat	United States, Canada, northern Mexico
*Procyon lotor* L., 1758	Racoon	Central and North America
*Rattus norvegicus* (Berkenhout, 1769)	Brown rat, Norway rat	Northeast China
*Sciurus carolinensis* (Gmelin 1788)	Grey squirrel, American grey squirrel, Eastern grey squirrel	Eastern United States and Canada
*Tamias sibiricus* (Laxmann, 1769)	Siberian chipmunk, common chipmunk	North Russia, China, Kazakhstan, Mongolia, North and South Korea
**Birds**		
*Branta canadensis* L., 1758	Canada goose	Canada, the Caribbean, Mexico, United States
*Oxyura jamaicensis* (Gmelin, 1789)	Ruddy duck	North America, the Caribbean, Andean regions of South America
*Psittacula krameri* (Scopoli, 1769)	Rose-ringed parakeet	Bening, Burkina Faso, Cameroon, Chad, Côte d'Ivoire, Dijibouti, Ethiopia, Gambia, Ghana, Guinea, Guinea-Bissau, Mali, Mauritania, Niger, Nigeria, Senegal, Somalia, Sudan, Togo, Uganda, Afghanistan, Bangladesh, Buthan, India, Nepal, Myanmar, Pakistan, Sri Lanka
*Threskiornis aethiopicus* (Latham, 1790)	African sacred ibis	Great part of Africa, Iraq, Kuwait
**Amphibians**		
*Lithobates catesbeianus* (Dubois, 2006)	American bullfrog	North America
**Reptiles**		
*Trachemys scripta* (Schoepff, 1792)	Slider turtle, yellow-bellied slider turtle	Mexico, United States

Native ranges were retrieved from the CABI invasive species compendium^68^.

To assist the prioritization of management strategies and policies, and to prevent future impacts of these IATV, this study aims to improve our understanding of the environmentally suitable areas for these species in Europe. We identify the areas where IATV potentially concentrate or are less present accounting for the areas where significant knowledge gaps exist. The specific factors determining the persistence of these terrestrial vertebrates in Europe are largely unknown, partly because they occupy wide and/or opportunistic niches and because previous studies are mostly local. Here we close this gap by modeling broad suitability areas for these IATV species at a continental scale based on a complete set of climate, land-use and additional habitat descriptors. We fit SDMs within Europe for each species adapting the hierarchical method suitable for invasive species particularities (weighting European pseudo-absences based on previously fitted global climatic models)^15^ and using exclusively open-access occurrence data from the Global Biodiversity Information Facility (GBIF)^24^. Predictions obtained from SDMs allow delimiting hotspots of IATV richness and devising a priority management area classification that besides considers the uncertainty of predictions. Quality and accuracy of GBIF data are often questioned; however, we use this uncertainty to identify areas where further data collection is urged as a management action to approach IATV threats. Our results may contribute to prioritize and focus strategies to reduce the detrimental impacts caused by IATV in Europe, and our methods could be applied to other areas and species worldwide.

## Results

European models (including climatic, land-use, accessibility, and other predictors) performed well for most species (Table 2; Supplementary Fig. S2.1.3), with an average of 91% of presences and 86% pseudo-absences correctly predicted. Model uncertainties fluctuated among species (CV_range_ = 0.09–0.70; Table2; Supplementary Fig. S3.3.1). Range filling (i.e. the proportion of reported presences over the binary potential suitable area for each species) varied widely among species, with values as low as < 0.1 for *L. catesbeianus* and *T. sibiricus*, and relatively high values (> 0.5) for *B. canadensis* and *Rattus norvegicus* (Table 2; Supplementary Fig. S2.1.4). Climatic predictors were the most relevant to predict environmental suitability in the European models for all species, with additional important variables for certain species, such as the fraction of urban areas for *T. sibiricus*, or roughness for *L. catesbeianus* (Supplementary Table S2.1.1). Global only-climatic models, fitted to weight the European pseudo-absences, correctly predicted on average 93% of the global presences (sensitivity) and 87% of the pseudo-absences (specificity) of IATV (Table 3; Supplementary Fig. S2.1.1). Model uncertainties varied among species, ranging from CV = 0.08 (*Oxyura jamaicensis*) to 0.63 (*N. vison*; Supplementary Fig. S2.1.2), being on average higher than in European models. As expected, climatic suitable areas (binary predictions based on global models) were larger than environmentally suitable areas (binary predictions based on European models; Supplementary Fig. S2.1.4). For some species such as *Neovison vison* or *B. canadensis*, climatic suitable areas were not predicted as fully environmentally suitable although some presences were reported in those only-climatic suitable areas (Supplementary Fig. S2.1.4).

**Table 2:** Results on the predictive ability of the European models fitted with *certain* datasets.

Species	n	TSS	Sensitivity	Specificity	Cut-off binary	Mean CV	Range filling
**Mammals**							
*Cervus nippon*	368	0.83	95.09	87.63	0.64	0.31	0.18
*Myocastor coypus*	2,444	0.63	82.03	81.41	0.58	0.56	0.47
*Neovison vison*	1,328	0.81	92.74	87.99	0.63	0.44	0.51
*Nyctereutes procyonoides*	422	0.72	92.12	80.38	0.58	0.42	0.14
*Ondatra zibethicus*	1,223	0.68	87.83	79.75	0.08	0.44	0.31
*Procyon lotor*	405	0.84	94.57	89.61	0.67	0.33	0.23
*Rattus norvegicus*	3,064	0.74	90.10	83.87	0.58	0.09	0.63
*Sciurus carolinensis*	631	0.93	95.51	97.29	0.61	0.37	0.54
*Tamias sibiricus*	71	0.86	100.00	86.00	0.41	0.25	0.03
**Birds**							
*Branta canadensis*	3,704	0.66	81.64	84.23	0.58	0.70	0.67
*Oxyura jamaicensis*	528	0.73	88.55	84.24	0.75	0.28	0.20
*Psittacula krameri*	564	0.74	88.45	85.11	0.41	0.50	0.21
*Threskiornis aethiopicus*	281	0.79	92.47	86.43	0.56	0.28	0.13
**Amphibians**							
*Lithobates catesbeianus*	51	0.88	96.08	91.89	0.58	0.56	0.04
**Reptiles**							
*Trachemys scripta*	1,210	0.73	86.03	86.69	0.69	0.32	0.40

*n* indicates the number of presence points used to fit the model. *TSS* is the true skill statistic. *Sensitivity* is the proportion of positives correctly predicted. *Specificity* is the proportion of absences correctly predicted. *Cut-off binary* shows the value of suitability (0–1) that maximized the sum of sensitivity and specificity and was used to convert the continuous prediction into binary. *Mean CV* is the mean coefficient of variation among models in the ensemble prediction standardized between 0 and 1. *Range filling* represents the fraction of the grid-cells classified as ‘presence’ in the binary map that overlapped with the observed records on species presences.

**Table 3:** Results on the predictive ability of the global models using the *certain* + *NA* dataset.

Species	n	TSS	Sensitivity	Specificity	Cut-off binary	Mean CV
**Mammals**						
*Cervus nippon*	457	0.91	98.02	92.92	0.44	0.55
*Myocastor coypus*	3,151	0.83	94.25	88.85	0.54	0.62
*Neovison vison*	2052	0.87	93.84	93.56	0.59	0.63
*Nyctereutes procyonoides*	689	0.88	96.92	91.56	0.49	0.54
*Ondatra zibethicus*	2,321	0.77	95.24	81.91	0.20	0.60
*Procyon lotor*	2,635	0.75	89.59	85.42	0.75	0.47
*Rattus norvegicus*	3,880	0.85	92.83	92.13	0.63	0.59
*Sciurus carolinensis*	2088	0.86	96.01	89.84	0.27	0.56
*Tamias sibiricus*	93	0.88	91.21	96.37	0.64	0.48
**Birds**						
*Branta canadensis*	22,953	0.73	86.11	86.66	0.73	0.37
*Oxyura jamaicensis*	13,030	0.70	89.99	80.31	0.30	0.08
*Psittacula krameri*	5,741	0.70	88.51	81.08	0.58	0.53
*Threskiornis aethiopicus*	2,694	0.77	93.52	83.48	0.50	0.10
**Amphibians**						
*Lithobates catesbeianus*	1937	0.76	92.56	83.73	0.44	0.46
**Reptiles**						
*Trachemys scripta*	2,863	0.79	92.44	86.68	0.49	0.57

*n* indicates the number of presence points used to fit the model. *TSS* is the true skill statistic. *Sensitivity* is the proportion of positives correctly predicted. *Specificity* is the proportion of absences correctly predicted. *Cut-off binary* shows the value of suitability (0–1) that maximized the sum of sensitivity and specificity (TSS) and was used to convert the continuous prediction into binary. *Mean CV* is the mean coefficient of variation among models in the ensemble prediction standardized between 0 and 1.

**Figure 1:**
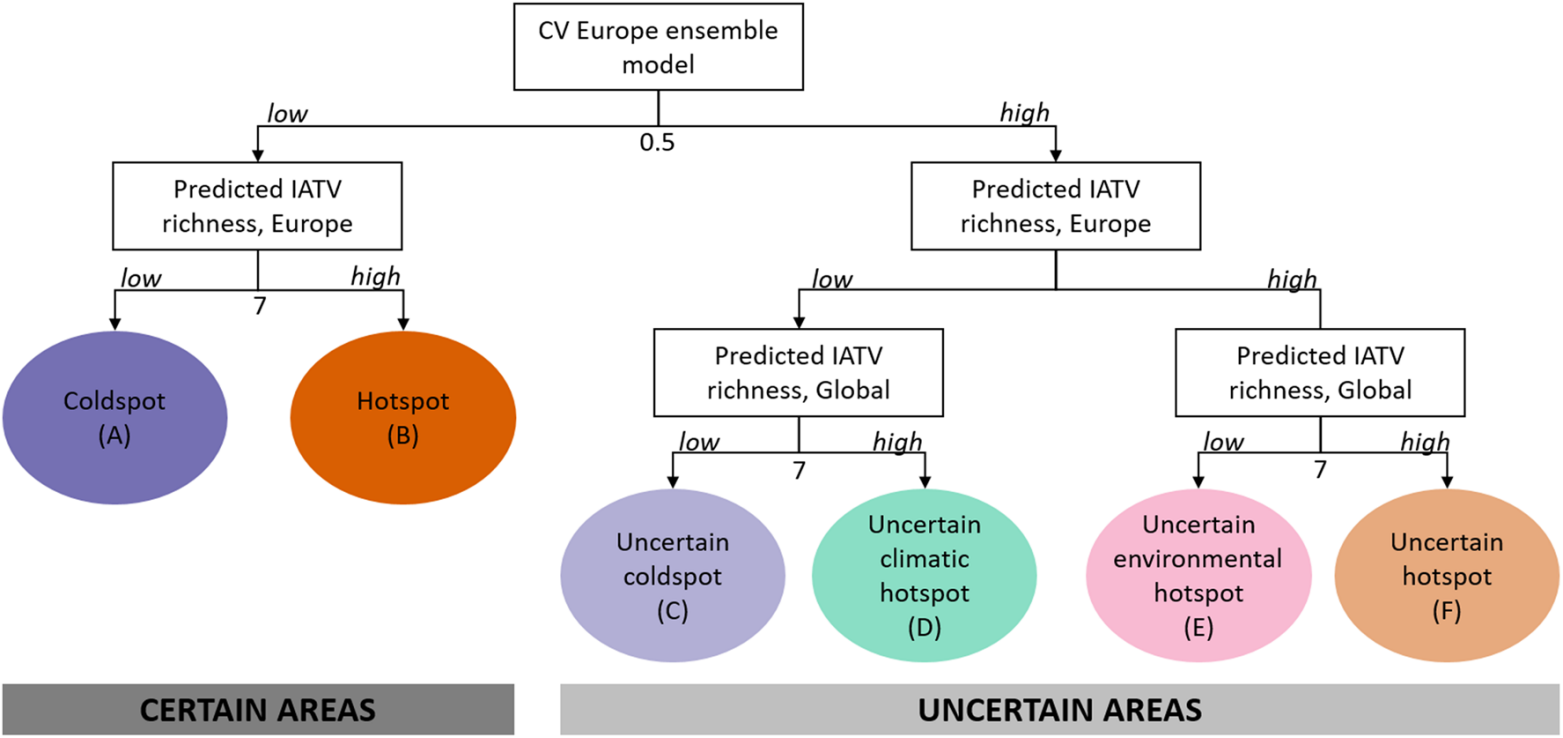
Classification tree applied to determine the category of each grid-cell within the ‘priority management areas’ classification. *CV Europe ensemble model* is the average of the coefficient of variation of the 15 IATV European ensemble models (including all type of predictors). *Predicted IATV richness, Europe* is the sum of all binary predictions of the 15 IATV European ensemble models. *Predicted IATV richness, Global* is the sum of all binary predictions of the 15 IATV global ensemble models (including only climatic predictors). Thresholds to determine *high* and *low* values are the central values for each variable (CV = 0.5; predicted IATV richness = 7).

**Figure 2:**
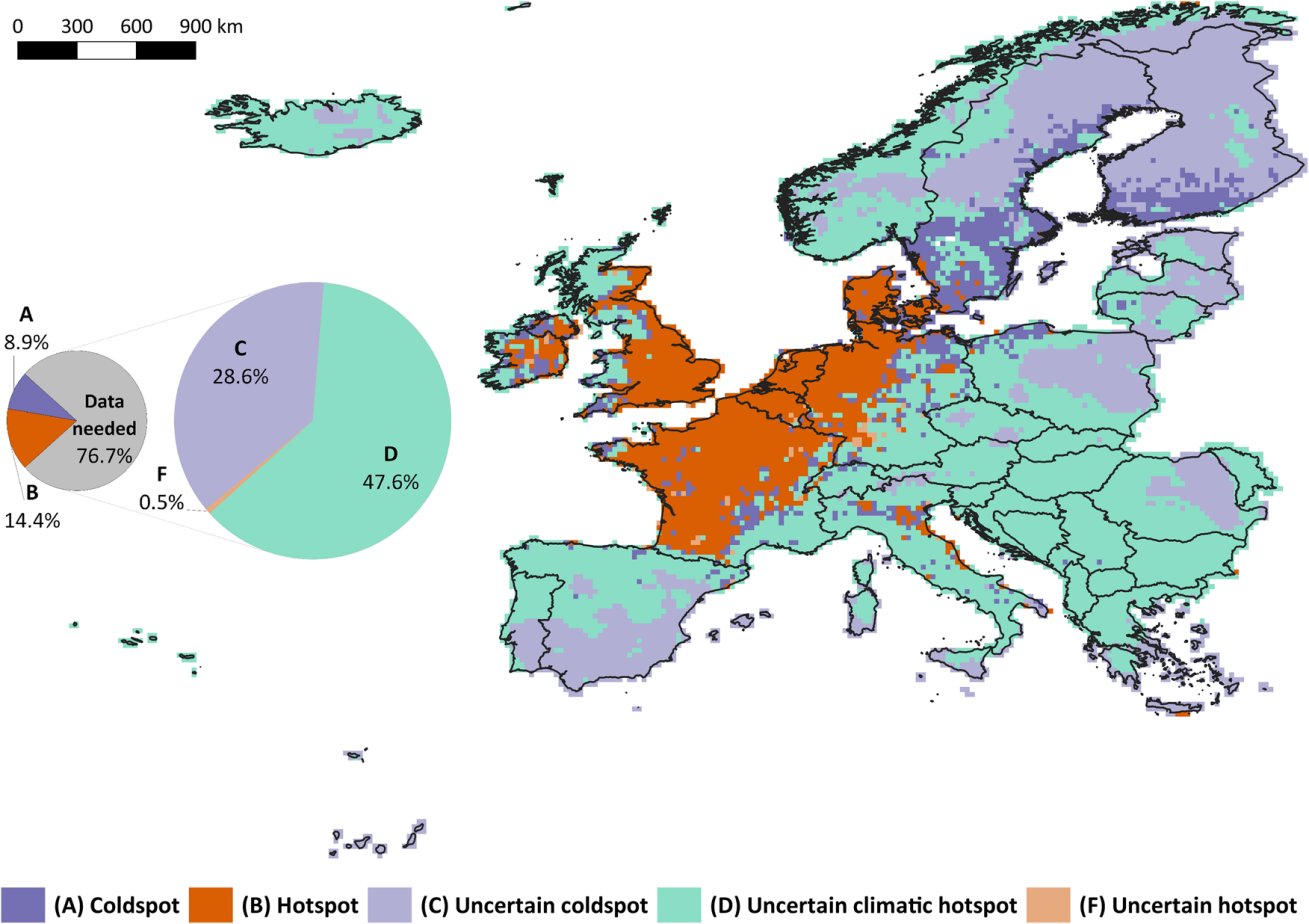
Priority management areas obtained from the application of the classification criteria described in Fig. 1, based on Supplementary Figs. S2.2.1, S2.2.2 and S3.3.1. The pie-chart to the left represents the proportion of grid-cells that belong to each class after aggregating all the categories under the ‘uncertain areas’ group, i.e. zones C to F (grey area). The pie-chart to the right represents the percentage of grid-cells within each category within ‘uncertain areas’. No grid-cell was classified as E (uncertain environmental hotspot). This figure was generated with QGIS v.3.2.3^66^ (www.qgis.org).

Predictions of the European models showed the maximum predicted IATV richness per grid-cell in the northwest part of continental Europe and the British Islands. Minimum predicted IATV richness values occurred in eastern Europe, northern Scandinavia, and Iceland (Supplementary Fig. S2.2.1). Areas of high IATV richness defined by only-climatic global predictions were wider, as expected from less restrictive models (Supplementary Figs. S2.2.1 and S2.2.2). Based on the uncertainty criteria, measured by the average CV of all-species European ensemble predictions, we defined two groups of areas within Europe, certain (low CV values; A–F, Fig. 1) and uncertain (high CV values; C–F, Fig. 1). Within the first group, areas of high predicted IATV, ‘hotspots’ (B), covered about 14% of Europe, and were mostly concentrated in central north-western Europe (Fig. 2). Several ‘coldspot’ areas of IATV were determined (A), conforming ~ 9% of the grid-cells and mostly located in southern Sweden and Finland, northern Germany, and scattered patches in central France and Ireland (Fig. 2). Within uncertain areas, areas where European and global predictions agreed conformed either ‘uncertain hotspots’ (0.5% of study area; F, Fig. 2) or ‘uncertain coldspots’ (29% of study area; C, Fig. 2). Around 48% of Europe (east and south of continental Europe) was predicted as climatically suitable (global model) for numerous IATV species, although this was not consistent with the European models, predicting low IATV richness; therefore, it was classified as ‘uncertain climatic hotspots’ because it would be classified as hotspot only attending to climatic criteria (D, Fig. 2). No grid-cell was identified as an ‘uncertain environmental hotspot’ (E), characterized by high IATV richness according to the European model (all environmental predictors) and low IATV richness as for the global model (only climatic predictors; Fig. 1). Uncertainties associated with dissimilar environmental conditions (measured by MESS) and with GBIF spatial bias (measured by half-ignorance index) were on average higher within ‘uncertain coldspots’ and ‘uncertain climatic hotspots’, being minimum within ‘hotspots’ (Table 4). These analyses overall revealed that the predictions in eastern Europe, north of Scandinavia, Iceland and the Iberian Peninsula would be more uncertain due to dissimilar environmental conditions, with differences among species (Supplementary Fig. S3.1.1). Looking at the GBIF spatial bias, we found eastern Europe, Portugal, northern Fennoscandia and Iceland are the most poorly sampled areas (Supplementary Fig. S3.2.1.1).

**Table 4:** Summary statistics of the different sources of uncertainty, associated with the environmental predictors (*MESS*, Multivariate Environmental Similarity Surfaces, expressed as the proportion of species present in each grid-cell presenting dissimilar environments); the occurrence data (*Ignorance index*, half-ignorance index per grid-cell calculated as in Supplementary Eq. S1; higher values indicate less occurrence data); and the variability of predictions (CV, average coefficient of variation per grid-cell over all-species ensemble predictions).

Zones	MESS			Ignorance index			CV		
	Mean	Min	Max	Mean	Min	Max	Mean	Min	Max
A. Coldspot	0.30	0.00	0.80	0.44	0.02	1.00	0.45	0.29	0.50
B. Hotspot	0.15	0.00	0.87	0.24	0.00	1.00	0.31	0.00	0.50
C. Uncertain coldspot	0.61	0.20	1.00	0.70	0.02	1.00	0.67	0.50	0.97
D. Uncertain climatic hotspot	0.52	0.00	1.00	0.68	0.01	1.00	0.66	0.50	0.98
E. Uncertain hotspot	0.28	0.07	0.67	0.39	0.02	0.92	0.53	0.50	0.63

Mean, minimum (Min) and maximum (Max) values per zone are reported.

We decided to include occurrence data of unknown spatial uncertainty (as defined by GBIF) in global models to incorporate undersampled regions of the world (*certain* + *NA* dataset; Supplementary Fig.S1.1). Using the *certain* + *NA* dataset led to slightly less accurate and more uncertain results, with small differences in spatial predictions except for birds, compared to the alternative fitted using only occurrences with uncertainty appropriate for our spatial resolution (*certain* dataset; Supplementary Table S2.1.2, Supplementary Figs. S2.1.6–S2.1.7). However, the derived European models were essentially as good as those based on global models fitted with *certain* datasets (Table 2 vs. Supplementary Table S2.1.3), with differences among species. Continuous environmental suitability predictions were also very similar (Bhattacharyya distances < 0.08; Supplementary Table S2.1.3 and Supplementary Fig. S2.1.9). On the other hand, we prioritized precision in European models, thus we removed occurrence data of unknown spatial uncertainty (*certain* dataset). Adding uncertain observations (*NA* + *certain* dataset*)* to fit European models resulted in less accurate and more uncertain models (Supplementary Table S2.1.4), with suitability predictions relatively dissimilar to the main version (fitted with only *certain* datasets at the European level, but including *NA* observations globally; Bhattacharyya distances < 0.40; Supplementary Table S2.1.4 and Supplementary Fig. S2.1.11). Overall, using the *certain* + *NA* dataset instead of the *certain* one to fit European models did not reduce uncertainties associated with dissimilar environmental conditions, except for three species of birds (Supplementary Figs. S3.1.1 and S3.1.2).

## Discussion

We contributed with a spatial approach to forecast potential environmentally suitable areas for 15 of the most harmful IATV in Europe to assist in prioritizing decision-making on the management of these species. Our approach enabled the identification of certain hotspots in terms of predicted species richness, where the implementation of control measures is crucial. We also incorporated model uncertainties to identify where improved species monitoring efforts are necessary, and assessed additional sources of uncertainty to support our results. Northwestern Europe accounts for the largest areas of potential IATV hotspots, but additional smaller hotspots are distributed over the study area. Nearly 77% of the continent requires further monitoring efforts to collect additional information on IATV presence that can improve model predictions on the potential distribution of these species and better inform management actions of prevention and control.

The main IATV hotspot in central north-west Europe would provide suitable environmental conditions for most of the studied species, including in areas of current unknown presences (B; Fig. 2). This hotspot is characterized by dense human populations, high socioeconomic development, and high levels of human disturbance, which are all factors typically associated with increased concentrations of IAS in general^25^. Previous research based on additional taxa and data sources also revealed the potential of these areas to concentrate alien species^26,27^. Additionally, a greater awareness of the problem of invasive species by the authorities and the general public might occur in these areas and result in more exhaustive dataset compilations from opportunistic citizen science data and standardized inventories. Rich and complete datasets on species presences could explain the high certainty of predictions in these areas (Table 4). The concentration of several invasive species in the same geographical area may add extra pressure on native ecosystems, and make any control program more challenging^28^. Firstly, different species have different habitat requirements, behaviours and ecology, and thus require different control measures that all together may be costly and logistically arduous. Second, IATV affect various socioeconomic sectors (cattle industry, croplands, cities) and natural systems (protected areas or species), which may cause conflicts of interests among stakeholders and thus, hindering the establishment of management priorities^29^. Third, high concentrations of IATV across national borders represent a significant challenge for control programs and transboundary policies and thus, requires cooperation between countries^30^. Considering all the potential negative impacts of IATV on native ecosystems, the economy and the management challenges that a high concentration of IATV would imply, prevention and early control should be prioritized within these areas. In this way, damages can be reduced and it could be possible to prevent potential reservoirs or stepping stones from source hotspots where IATV can expand into neighboring suitable areas^31^.

Only 9% of Europe was identified as coldspots for the IATV studied (A, Fig. 2) and mostly included areas in southern Fennoscandia. From socioeconomic and social-awareness perspectives, these areas are similar to those of IATV hotspots. However, Fennoscandia is less populated than most other hotspots in central Europe, and the climatic conditions are more extreme and less suitable for several species such as *C. nippon* or *L. catesbeianus* (Kaji et al*.*^33^; Yiming et al*.*^32^). In principle, less IATV species would imply lower impacts. However, ignorance related to GBIF datasets is higher within these areas than within hotspots, therefore greater caution is required (Table 4). Besides, these northern regions can host vulnerable native species and ecosystems sensitive to even few IATVs (e.g. predation of breeding wetland birds by *N. vison*^34^). Fewer IATV species makes control and eradication objectives a priori more approachable. Still, if relatively extreme climate conditions are limiting IATV expansion into these areas, caution is also required for the future effects of climate change. The potential warming of these areas could promote more favorable habitats for some IATV species and gradually become hotspots^35^.

Some other areas of Europe may also present favorable conditions to harbor large numbers of IATV; however, these remain unidentified due to different uncertainties, as suggested by the high variability within the predictions of our models. More than 75% of the studied area in Europe would benefit from additional occurrence data on IATV to provide improved information on their potential distribution, invasion risk, and subsequent detrimental impacts. We confirmed uncertain predictions within uncertain coldspots and uncertain climatic hotspots are mostly caused by a lack of data and, consequently, coverage of specific environmental conditions where it is hard to infer accurate predictions (Table 4). Within ‘uncertain’ areas, those presenting high climatic suitability but low environmental suitability deserve special attention (uncertain climatic hotspots; Fig. 2D). Although these areas have favorable climatic conditions for numerous species, non-climatic factors might be limiting the distribution of IATV, added to other possible causes such as dispersal limitations or low propagule pressure^36^. Caution is required for the potential changes of land-use that could create suitable environments for IATV and/or remove dispersal barriers for the expansion of IATV into climatically favorable areas. Additional data covering all environmental conditions in Europe could help to understand the on-going process in the uncertain areas (Supplementary Figs. S3.1–S3.8).

Although using exclusively GBIF data might limit predictive capabilities of the models, the use of publicly available and feasible occurrence data guarantees transparency and the possibility to improve models when more data become available, key for invasive species. New legislation and rankings of invasive species constantly appear, thus having a unified data source and a common analytical framework are key to guarantee long-term evaluations^37,38^. Using fragmented, dispersed, not-standardized, non-digitalized, and often not publicly available data hampers modelling initiatives at wide scales, hinders reproducibility, and impedes real comparison among similar studies. Consequently, we suggest as common practice the uploading of occurrence data from literature reviews into an established open-access occurrence dataset, such as GBIF^39^. Alternative initiatives to collect data on species’ presence over large spatial scales already exist and are downloadable upon request. However, these sources often focus on over-represented areas of EU and USA, or consider the occurrences separately in invasive and native ranges (e.g. CABI-ISC, https://www.cabi.org/isc; ISSG, https://www.issg.org; EASIN, https://easin.jrc.ec.europa.eu). We acknowledge our data comes from a snapshot of a continuously growing dataset repository relying on different levels of participation between countries and regions, which may potentially lead to geographic bias on IATV presence datasets. However, our analysis framework explicitly incorporates the identification of these areas as ‘uncertain’, i.e. where predictions are less solid, omission errors are more probable, and vice versa. Reinforcing the participation of non-EU countries in EU policies against IAS would enhance the success of control programs at continental levels (particularly in border countries) and it would contribute to data collection in unified initiatives like GBIF^40^. In general, the data coverage of GBIF in Europe is similar to other reference organisms such as CABI, which lacks point-occurrence information (Supplementary Table S3.2.2.1).

We acknowledge uncertainty is an inherent characteristic to all statistical models (Supplementary SI3). This uncertainly is particularly high at broad scales and when using data containing opportunistic records such as GBIF. However, the identified uncertainty can be seen as an advantage as it allows us identifying where greater support for continuous and intensive efforts on monitoring IATV is required^41^. We identified the lack of data in some regions as the main source of uncertainty, which implies not all the environmental conditions included in the models are equally covered (Supplementary Information S3). Another concern could arise from the predicted suitable ranges, which might potentially be overestimated, particularly by our global models. Nevertheless, this attribute is considered as an advantage to predict potential areas of invasion where false positives are preferred over false negatives in management actions^42^. Moreover, these predictions were exclusively used to weight European pseudo-absences, and to confirm European predictions within uncertain areas. We aimed a trade-off by including an expanded dataset of species presences to fit the global models and a more restricted dataset for the European models. This way, we confirmed that including *NA* observations in the global models did not compromise model accuracy and spatial predictions. On the contrary, including *NA* observations in the European models led to poorer results. Overall, our research approach delimitates risk areas on a broad scale while implementing management and research recommendations, and sets a baseline for research on the future expansion of IATV.

Future scenarios on climate and land-use changes are expected to influence the invasion capacity of the species through synergistic processes^43,44^. Considering available data on climate and land-use changes are mostly available at low resolutions^45^, these could be directly incorporated into our modeling to strengthen the forecasting of plausible scenarios and to interpret alternative invasion processes, either facilitating or constraining IATV expansion. These initiatives would be precious to account for the future impacts of IATV so that decision-making in management strategies could adjust to coming changes.

European regulations against invasive species highlight the need for international transboundary cooperation to achieve prevention and control successfully within member states^46^. However, IATV detection and study is still very fragmented in Europe^21^, which hinders collaboration between different countries and the implementation of common transboundary strategies to solve shared conservation problems. In addition, collaborations often exclude non-EU countries with similar concerns on IATV management. Our research is a valuable initiative to warn about the dimension of the IATV problem at a continental level, highlighting the ignorance on their potential spatial distribution. Field studies, citizen-science initiatives and the promotion of open-access data availability are all fundamental to obtain the best possible quality data and to reduce the uncertainties here identified. Our modelling approach was specifically developed for invasive species and proposes a framework to provide results in a straightforward and replicable fashion, applicable to other areas. It also enables predictions on future potential suitable areas for these species under different climatic and land-use change scenarios.

## Methods

**Figure 3:**
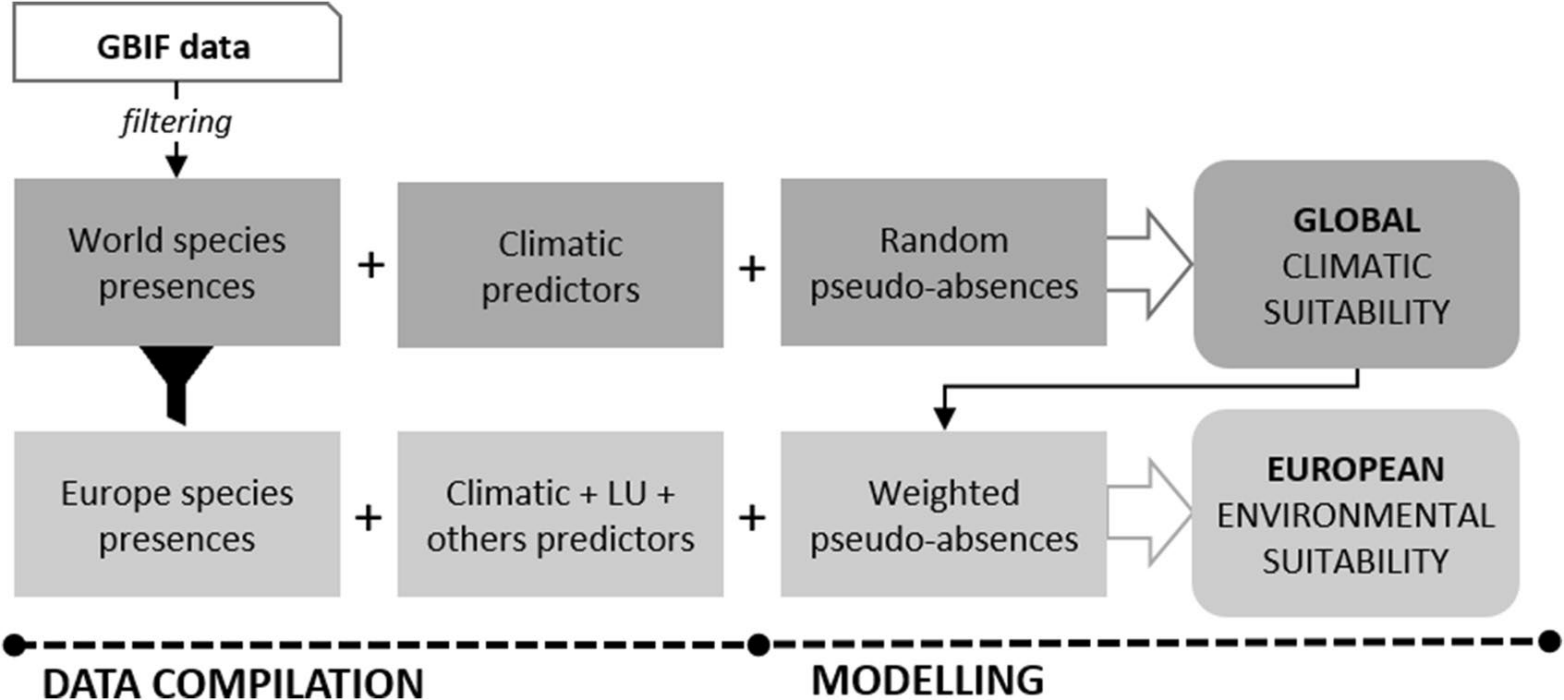
Workflow of the methods used to obtain global climatic and European environmental suitability for each of the 15 invasive alien terrestrial vertebrates (IATV) of study.

We followed a SDM approach devised for invasive species based on presence-only data^15^. This approach consisted of the fitting, for each IATV species, firstly a global model, and second a European model within the invaded area of interest (Fig. 3).

We considered Europe as the area comprised of the 27 European Union countries, Great Britain, Norway, Switzerland, Iceland, the Balkan countries, and the European microstates (Supplementary Table S1.1). Global models approximated the global climatic niche of each species by including the native and invaded ranges to account for the non-conservation of the niche typical of IAS^47^. To correct for the lack of equilibrium with the recipient environment, the European models incorporated the output of the global model to weight the reliability of each pseudo-absence. European models estimated the realized regional niche and represented current environmental suitability according to the information available, instead of a comprehensive description of the ecological niche of each species^14^. We computed global and European models using a grid size of 0.25° × 0.25° (c. 30 × 30 km), corresponding to the minimum available resolution of the predictor datasets on land use. All analyses were made in R^48^.

### Data compilation

We assumed the climate was the main factor limiting global species’ distribution, whereas, at the European scale, additional factors shaped their probability of presence^13^. Therefore, we selected only climatic variables for the global models (CHELSA database^49^) and added general habitat descriptors to the European models, including variables on land use (land-use harmonization project^45^), water availability^50,51^, distance to the coast, topography^52^, and accessibility to major cities (distance based^53^; Supplementary Tables S1.2 and S1.3). When required, we averaged raster predictors to the adopted pixel resolution of 0.25° × 0.25° (Supplementary Box S1.1 and Supplementary Table S1.4). We used the same variables for all the species to follow a parallel modelling approach, and because inferring specific causality at this scale and resolution is unrealistic. Predictors exhibiting multicollinearity (variance inflation factor, VIF > 4) were excluded from all models (‘vifstep’ function, *usdm* package^54^), which resulted in a final set of 8 and 18 for the Global and European model, respectively (Supplementary Table S1.3).

We obtained occurrences of the 15 IATV included in the DAISIE list ‘100 of the Most Invasive Alien Species in Europe’, an expert-based ranking aimed to cover the most harmful IAS in Europe. We downloaded data from GBIF^24^ (Supplementary Table S1.5) using the *rgbif* package^55^. We selected all the available georeferenced observations collected worldwide from human or machine observations (e.g. camera traps). We also filtered our data using the GBIF field ‘uncertainty in meters’, and selected records with an uncertainty ≤ 15,000 m to match the circumference radius of c.30 km of our grid-cell size. Excluding uncertain records to fit the models reduced the inclusion of environmental conditions not associated with the species. However, entries containing unknown (NA) ‘uncertainty in meters’ are numerous in GBIF, which may be caused by skipping the filling of this information. The inclusion of these observations increases the number and coverage of presence records, namely for the less-sampled regions in Africa or Asia^56^ (Supplementary Fig. S1.1 and Supplementary Table S1.5). We incorporated these records in the global model to maximize the estimated global climatic niche and to capture the greatest number of regions where the species are present (*certain* + *NA* dataset; N_range_ = 93–22,953 for the 15 species; Table 3) but removed NA records to fit European models (*certain* dataset; N_range_ = 51–3,704; Table 2), where we prioritized precision. Using the package *CoordinateCleaner*^57^*,* we further removed common spatial errors (i.e. country centroids, equal longitude-latitude observations, GBIF headquarters, biodiversity institutions and zero coordinates). Finally, only one observation per grid-cell was retained to control for pseudo-replication and to reduce the spatial bias of GBIF^58^. To test the influence in the results of using *certain* and *certain* + *NA* datasets as explained, we also run the models using *certain* datasets to fit global models, and *certain* + *NA* datasets to fit the European model (Supplementary SI2).

### Modelling

#### Handling SDM pseudo-absences

For each species, we first ran a global model randomly selecting pseudo-absences (N = 20,000) within the entire global land surface (excluding Antarctica). We also randomly located pseudo-absences (N = 5,000) over Europe in the European model but weighted them by the climatic suitability obtained from the global model. The lower the climatic suitability in a given location, the higher the probability a pseudo-absence tended to be a real absence, and vice versa. We calculated pseudo-absence weights using an inverse logistic transformation^15^ (Eq. 1):

1 $$Weight \left( x \right) = \frac{1}{{1 + \left( {\frac{projG\left( x \right)}{{projG\left( x \right) - 1}}} \right)^{2} }},$$

where *Weight(x)* is the weight of the pseudo-absence in the location *x*, and *projG(x)* is the global model prediction in *x*. If *projG(x)* = 1 then *Weight(x)* = 0.

#### Fitting species distribution models (SDMs)

We fitted SDMs using five algorithms in the *BIOMOD2* package (version 3.3-7^59^), generalized linear model (GLM), generalized additive model (GAM), flexible discriminant analysis (FDA), generalized boosting model (GBM) and maximum entropy (MAXENT. Phillips; Supplementary Table S1.6). Each model ran three times for each algorithm using alternative sets of random pseudo-absences. To validate the model performance, we applied a cross-validation procedure using 70% of the data for the model training and 30% for the model evaluation^60,61^. The complete process resulted in 60 models per species and spatial setting (i.e. global or European). The resultant models produced from the different algorithms were used to build an ensemble model by applying the committee averaging method^60^ (predictions close to 0 or 1indicate models agree to predict 0 and 1, respectively. Predictions of ~ 0.5 indicate half of the models predict 1 and the other half 0). Whenever possible, only individual models with a good predictive performance were selected, measured by the true skill statistic (TSS). This measure combines the model ability to predict presences correctly (sensitivity) and pseudo-absences (specificity) independently of the prevalence^62^ (TSS ≥ 0.7). If none of the models concurred with the minimum threshold value, we selected the best available models (10% top quantile) to ensure the production of one ensemble model for each species and spatial setting. For each ensemble model, we converted the resulting continuous predictions (between 0 and 1) into a binary classification that approximated the suitable area of the species using a threshold that maximized the TSS^63^. We also calculated the coefficient of variation (CV) and the predictive performance of the ensemble models as specificity, sensitivity and TSS. As a proxy of the distance to pseudo-equilibrium, we calculated the range filling of each IATV as the proportion of pixels of reported presences that overlapped the predicted binary presence obtained from the European model^15^.

### Multi-species summary

Combining all the binary predictions of each European model output, we estimated the potential IATV richness as the total number of species that scored a presence in each grid-cell, which indicated the potential number of species that would find suitable conditions to persist^27^. Additionally, we implemented a classification system per grid-cell to define general priority management areas (Fig. 1) from three indicators: (i) the uncertainty of the predictions in the European models, (ii) the invasive species richness as predicted by the European models, and (iii) the invasive species richness as predicted by the global models. The uncertainty of predictions was calculated as the average of the coefficients of variation (CV) of the European ensemble models for each species. We categorized each of the three indicators into two groups (high and low) using the central value of its range, which was seven for species richness in both the European and Global model predictions (range between 0 and 15), and 0.5 for CV (range between 0 and 1). Grid-cells scoring a high CV (> 0.5) depicted different suitability values across algorithms, pseudo-absences and cross-validation runs, whereas low CV values indicated agreement among model predictions.

We considered the most reliable results as those of low average CV over all the species (< 0.5, ‘certain areas’). In this group (categories A and B), the areas with a high predicted IATV richness (IATV hotspots; Fig. 1B) were considered of high ecological and socioeconomic concern given their potential to harbor a higher number of IATV species and, consequently, to receive increased impacts (e.g. predation or spread of diseases to native fauna, damages to croplands or forestry). The opposite applied to IATV coldspots (areas with low predicted IATV richness; Fig. 1A). Conversely, we considered that a high mean CV required cautious interpretation due to the disagreement among the different model outputs, regardless of the predicted IATV richness (categories C to F, Fig. 1). To better characterize these uncertain areas, we further classified them considering the predicted IATV richness calculated from the global model. Where global and European predictions agreed and IATV richness values were high, we assumed a high probability of being an IATV hotspot (uncertain hotspots, Fig. 1F). The areas where both predictions were low would be most likely coldspots (uncertain coldspots, Fig. 1C). Sometimes predicted invasive species richness disagreed between global and European models. In that case, the areas with high species richness as predicted by the European models but low according to the global predictions would indicate unfavorable climatic conditions for many species. However, other local factors could facilitate the establishment of numerous IATV (e.g. urban areas, uncertain environmental hotspots, Fig. 1E). Alternatively, areas of low predicted IATV richness as predicted by the European models but high according to the global ones would imply potential favorable climatic conditions for numerous IATV. In this case, the non-climatic conditions of these areas could be unsuitable (uncertain climatic hotspots, Fig. 1D).

### Sources of uncertainty

To identify further sources of uncertainty besides those related to the parametrization of the model, we measured the uncertainty of extrapolating model predictions to environmental conditions not covered by the fitted model by calculating the Multivariate Environmental Similarity Surfaces (MESS) in R-package *dismo*^64^. Additionally, we computed ignorance maps (~ the inverse of the number of occurrences per reference group per grid cell) using taxonomical families as reference groups, which are useful tools to differentiate non-sampled areas from those containing real absences^65^. Ignorance maps assume the deficiency of reports of any species from a reference taxonomic group at a particular location is likely caused by a lack of observers rather than to the total absences of the species. Assuming equal sampling perception of native and invasive species by observers might be misleading because invasive species may be particularly well sampled in an area where a specific management project has been conducted. However, we used these maps to illustrate the spatial bias of the GBIF occurrence data and to help interpreting results (see Supplementary Information S3 for further details).

## Supplementary information

Supplementary information

## Data Availability

The original datasets are freely available on the sources mentioned in the text. Filtered versions of these data are available from the corresponding author on request. A complete code of the modelling procedure is available at [https://github.com/esterpolaina/Current_IATV_distribution].
