# Supplementary material for: Identifying hotspots of invasive alien terrestrial vertebrates in Europe to assist transboundary prevention and control
Source: Sci Rep. 2020 Jul 15;10:11655. doi: 10.1038/s41598-020-68387-3 (PMC7363869; doi:10.1038/s41598-020-68387-3)
Supplement: Supplementary file 1 — Supplementary information [file 41598_2020_68387_MOESM1_ESM.pdf]

Supplementary Information (SI)

Identifying hotspots of invasive alien terrestrial vertebrates in Europe to assist transboundary prevention and control

Ester Polaina, Tomas Pärt and Mariano R. Recio*

Authors' affiliations

Ester Polaina

Department of Ecology, Swedish University of Agricultural Sciences, Uppsala, Sweden

Tomas Pärt

Department of Ecology, Swedish University of Agricultural Sciences, Uppsala, Sweden

Mariano R. Recio*

mariano. recio@gmail.com

Department of Ecology, Swedish University of Agricultural Sciences, Uppsala, Sweden

Departamento de Biología y Geología, Física y Química Inorgánica, Universidad Rey Juan Carlos, ESCET, Móstoles, Madrid, Spain

*Corresponding author.

INDEX

SI 1: Description of data used to fit invasive species distribution models (SDM)..4

Table S1.1. List of countries included in the study area and their affiliation to the European Union (EU). .4

Table S1.2. List of all the initial predictors considered. 5

Table S1.3. List of non-collinear variables included in global and European models (VIF ≤ 4) ………… 8

Text Box S1.1 Methods to upscale predictors...8

Table S1.4. Results of the Spearman's correlation tests. 9

Figure S1.1. Global presences per grid-cell retrieved from GBIF and filtered a...10

Table S1.5. Number of observations directly retrieved from GBIF, and *certain/certain+NA* datasets.......10

Table S1.6. Algorithms selected to fit individual species' models. 11

SI 2: Complementary results...12

SI 2.1 Individual species models 12

a) Models included in the main text..12

Figure S2.1.1. Climatic suitability predictions obtained from the global ensemble SDMs 12

Figure S2.1.2. Coefficients of variation of the climatic suitability predictions. 13

Table S2.1.1 Variable importance according to the European models for each species. 14

Figure S2.1.3. Environmental suitability predictions obtained from the European ensemble SDM 15

Figure S2.1.4. Overlapped results of the binary predictions (global and European) and the presence data 16

b) Additional models considering different datasets of IATV presences...17

b.1) Using the *certain* dataset in the global and European models...17

Table S2.1.2. Predictive accuracy of the global models using the *certain* dataset.........................18

Figure S2.1.5. Climatic suitability predictions obtained from the global ensemble SDMs 19

Figure S2.1.6. Coefficients of variation of the climatic suitability predictions 20

Figure S2.1.7. Measures of dissimilarity per grid-cells (Bhattacharyya distance) between global predictions 21

Table S2.1.3. Predictive accuracy of the European models using *certain* datasets (global models fitted with *certain* datasets)...22

Figure S2.1.8. Environmental suitability predictions obtained from the European ensemble SDM 23

Figure S2.1.9. Measures of dissimilarity per grid-cells (Bhattacharyya distance) between European predictions 24

b.2) Using the *certain+NA* datasets in the global and European models 25

Table S2.1.4. Predictive accuracy of the European models using the *certain+NA* datasets 25

Figure S2.1.10. Environmental suitability predictions obtained from the European ensemble SDMs 26

Figure S2.1.11. Measures of dissimilarity per grid-cells (Bhattacharyya distance) between European predictions 27

SI 2.2 Multi-species summary 28

a) Models included in the main text..28

Figure S2.2.1. Predicted richness of invasive alien terrestrial vertebrate (IATV) derived from the European models...28

Figure S2.2.2. Predicted richness of invasive alien terrestrial vertebrate (IATV) derived from the global models 28

b) Additional models considering different datasets of IATV presences...29

b.1) Using the *certain* datasets in the global and European models...29

Figure S2.2.3. Predicted richness of invasive alien terrestrial vertebrates (IATV) derived from the European models....29
Figure S2.2.4. Predicted richness of invasive alien terrestrial vertebrates (IATV) derived from the global models....29
Figure S2.2.5. Priority management areas obtained from the application of the classification criteria described in Fig.1 (main text)....30

b.2) Using the *certain+NA* datasets in the global and European models....31
Figure S2.2.6. Predicted richness of invasive alien terrestrial vertebrates (IATV) derived from the European models....31
Figure S2.2.7. Priority management areas obtained from the application of the classification criteria described in Fig.1 (main text)....31

SI 3: Sources of uncertainty....32
SI 3.1. Uncertainty associated with environmental predictors....32
Figure S3.1.1. Multivariate environmental similarity surfaces calculated from the *certain* datasets....33
Figure S3.1.2. Multivariate environmental similarity surface calculated from *certain+NA* datasets....34

SI 3.2. Uncertainty associated with occurrence data of species....35
SI 3.2.1 GBIF geographic bias....35
Table S3.2.1.1. Species used in this work, family to which they belong and count of species within the family that are present in Europe according to GBIF (www.gbif.org)....37
Figure S3.2.1.1 Ignorance maps representing the half-ignorance score per grid cell for the families of the 15 IATV....38
Figure S3.2.1.2. Ignorance maps representing the half-ignorance score per grid cell for 15 IATV. *Certain* datasets were used....39
Figure S3.2.1.3. Ignorance maps representing the half-ignorance score per grid cell for 15 IATV. *Certain+NA* datasets were used....40

SI 3.2.2 Additional data sources....41
Table S3.2.2.1. Number of countries where each IATV species is reported, according to GBIF and CABI....41

SI 3.3 Uncertainty associated with predictions variability – CV of ensemble predictions....42
Figure S3.3.1. Coefficients of variation of the environmental suitability predictions from the European ensemble SDMs for each IATV. Global *certain+NA* dataset, European *certain* dataset....42

Figure S3.3.2. Coefficients of variation of the environmental suitability predictions from the European ensemble SDMs for each IATV. Global and European *certain* datasets....43

Figure S3.3.3. Coefficients of variation of the environmental suitability predictions from the European ensemble SDMs for each IATV. Global and European *certain+NA* datasets....44

References....45

SI 1: Description of data used to fit invasive species distribution models (SDM)

Table S1.1. List of countries included in the study area and their affiliation to the European Union (EU).

Country	EU member
Albania	NO
Andorra	NO
Austria	YES
Belgium	YES
Bosnia and Herzegovina	NO
Bulgaria	YES
Croatia	YES
Czech Republic	YES
Denmark	YES
Estonia	YES
Finland	YES
France	YES
Germany	YES
Greece	YES
Hungary	YES
Iceland	NO
Ireland	YES
Isle of Man	NO
Italy	YES
Latvia	YES
Liechtenstein	NO
Lithuania	YES
Luxembourg	YES
Malta	YES
Moldova	NO
Montenegro	NO
Netherlands	YES
Norway	NO
Poland	YES
Portugal	YES
Romania	YES
Serbia	NO
Slovakia	YES
Slovenia	YES
Spain	YES
Sweden	YES
Switzerland	NO
North Macedonia	NO
United Kingdom	NO

Table S1.2. List of all the initial predictors considered when estimating the 15 invasive alien terrestrial vertebrates (IATV) environmental suitability, using an SDM approach. Only variables of reduced multicollinearity were included in the final models (see Table S1.3).

**Predictor**	**Original class**	**Units**	**Year**	**Original resolution**		**Domain**		**Source**	**Version**	**Reference**
				Spatial	Temporal	Spatial	Temporal			
Climate										
Annual Mean Temperature – *BIO1*		°C	1979-2013	30" x 30"	Annual	Global	1979-2013	CHELSA -Climatologies at high resolution for the Earth's land surface areas (http://chelsa-climate.org/)	1.2	Karger et al (2017)[1]
Mean Diurnal Range (Mean of monthly (max temp - min temp) – *BIO2*		°C								
Isothermality *((BIO2/BIO7) (* 100))* – *BIO3*		%								
Temperature Seasonality *(standard deviation *100)* – *BIO4*		%								
Max Temperature of Warmest Month – *BIO5*		°C								
Min Temperature of Coldest Month – *BIO6*		°C								
Temperature Annual Range *(BIO5-BIO6)* – *BIO7*		°C								
Mean Temperature of Wettest Quarter – *BIO8*		°C								
Mean Temperature of Driest Quarter – *BIO9*		°C								
Mean Temperature of Warmest Quarter – *BIO10*		°C								
Mean Temperature of Coldest Quarter – *BIO11*		°C								
Annual Precipitation – *BIO12*		mm								
Precipitation of Wettest Month – *BIO13*		mm								
Precipitation of Driest Month – *BIO14*		mm								
Precipitation Seasonality (Coefficient of Variation) – *BIO15*		%								
Precipitation of Wettest Quarter – *BIO16*		mm								
Precipitation of Driest Quarter – *BIO17*		mm								
Precipitation of Warmest Quarter – *BIO18*		mm								
Precipitation of Coldest Quarter – *BIO19*		mm								

Predictor	Original class	Units	Year	Original resolution		Domain		Source	Version	Reference
Land use										
Forested primary land	Forested primary land	Fraction of grid cell	2015	0.25° x 0.25°	Annual	Global	850-2015	Land use harmonization2 (http://luh.umd.edu/data.shtml)	LUH2 v.2h	Hurtt et al (2011)[2]
Non-forested primary land	Non-forested primary land									
Potentially forested secondary land	Potentially forested secondary land									
Potentially non-forested secondary land	Potentially non-forested secondary land									
Urban	Urban									
Managed pasture	Managed pasture									
Rangeland	Rangeland									
Croplands	c3 annual crops, c3 perennial crops, c4 annual crops, c4 perennial crops, c3 nitrogen-fixing crops									
Water availability										
River density		km/grid-cell		10 m (vector)		Europe		Natural Earth (https://www.naturalearthdata.com/downloads/10m-physical-vectors/10m-rivers-lake-centerlines/)	4.1.0	Natural Earth (2018)[3]
Water bodies	Water bodies	ha/grid-cell	2018	100 x 100 m		Europe	2017-2018	Corine Land Cover (https://land.copernicus.eu/pan-european/corine-land-cover/lcc-2012-2018)	20b2	EEA (2018)[4]
Distance to the coast	Distance to the coast	km						Calculated as the distance from the centroid to closest coastline		
Topography										
Elevation		m	1996	30" x 30"		Global		GTOPO 30		LP DAAC (2004)[5]
Roughness		m	1996	30" x 30"		Global		GTOPO 30		LP DAAC (2004)[5]
Accesibility										

Predictor	Original class	Units	Year	Original resolution	Domain	Source	Version	Reference
Travel time to major cities (>50,000 people)		km	2000	30" x 30"	Global	Travel time to major cities: A global map of accesibility. Joint Research Center. The European Comission's science and knowledge service (https://forobs.jrc.ec.europa.eu/products/gam/download.php)		Nelson (2008)[6]

Table S1.3 List of non-collinear variables included in global and European models (VIF ≤ 4). Crosses indicate in which model they are included.

**Predictor**	Global model	European model
Climate		
Mean Diurnal Range	x	
Isothermality	x	x
Temperature Seasonality		x
Mean Temperature of Wettest Quarter	x	x
Mean Temperature of Driest Quarter	x	
Precipitation of Driest Month	x	
Precipitation Seasonality	x	x
Precipitation of Warmest Quarter	x	x
Precipitation of Coldest Quarter	x	x
Land use		
Forested primary land		x
Non-forested primary land		x
Potentially non-forested secondary land		x
Urban		x
Managed pasture		x
Rangeland		x
Croplands		x
Water availability		
River density		x
Water bodies		x
Distance to the coast		x
Topography		
Roughness		x
Accessibility		
Travel time to major cities (>50,000 people)		x

Text Box S1.1 Methods to upscale predictors

In order to obtain values as similar as possible to the original sources and resolutions, we upscaled the raster variables available at finer resolutions (see Table S1.2) using two different techniques. In our case, the limiting factor when selecting the working spatial resolution was the land-use data [2], which was only available at 0.25 x 0.25° resolution at the time of the analyses; therefore, some predictors were upscaled into this resolution. We tried a bilinear interpolation technique and the calculation of the average value of the grid-cells contained in the larger grid-cell. We checked for the resampling technique that, on average, best retained the original values using a Spearman's correlation test. The averaged values exhibited higher

correlation values with the original values, thus we selected this output instead of the resulting from the bilinear interpolation (Table S1.4).

Table S1.4. Results of the Spearman's correlation tests between the values of the original raster predictors and those resulting from applying a bilinear interpolation (*interpolation*) and an averaging resampling (*average*), respectively. These resampling methods were applied over the grid-cells contained within each 0.25 x 0.25° of the target grid-cells.

**Predictor**	**Spearman's ρ**	
Climate	Interpolation	Average
Annual Mean Temperature	0.998	0.999
Mean Diurnal Range	1.000	1.000
Isothermality	1.000	1.000
Temperature Seasonality	1.000	1.000
Max Temperature of Warmest Month	0.996	0.997
Min Temperature of Coldest Month	0.999	1.000
Temperature Annual Range	1.000	1.000
Mean Temperature of Wettest Quarter	0.990	0.991
Mean Temperature of Driest Quarter	0.996	0.997
Mean Temperature of Warmest Quarter	0.996	0.997
Mean Temperature of Coldest Quarter	0.999	0.999
Annual Precipitation	0.994	0.994
Precipitation of Wettest Month	0.992	0.993
Precipitation of Driest Month	0.996	0.997
Precipitation Seasonality	0.996	0.997
Precipitation of Wettest Quarter	0.992	0.993
Precipitation of Driest Quarter	0.997	0.997
Precipitation of Warmest Quarter	0.991	0.992
Precipitation of Coldest Quarter	0.993	0.996
Topography		
Elevation	0.973	0.979
Accessibility		
Distance to the closest major city (>50,000 people)	0.961	0.973

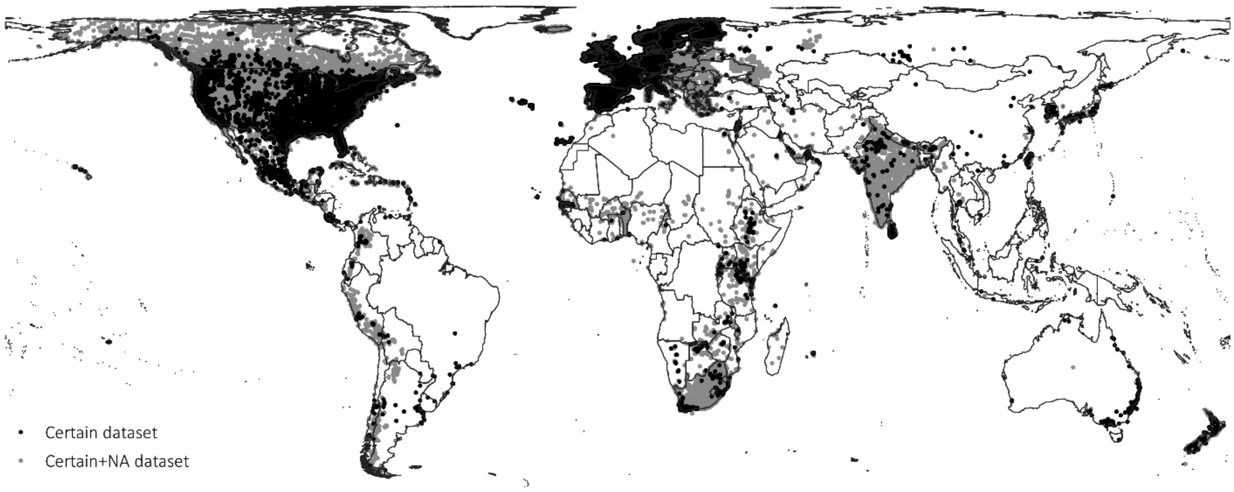

Figure S1.1. Global presences per grid-cell retrieved from GBIF and filtered as explained in the main manuscript. The *certain* dataset shows the selection of records with an uncertainty ≤15,000 m (i.e. 15 km, expressed as the radius of the circumference that matches our spatial resolution of c.30 km grid-cell size). The *certain+NA* dataset adds to *certain* dataset data of unknown values of coordinates uncertainty. Data downloaded in February 2019. This figure was generated with QGIS v.3.2.3[7] (www.qgis.org).

Table S1.5. Number of observations directly retrieved from GBIF (*Occur* = Occurrences). *Dataset* represents the actual number of points included in the models after filtering the original occurrences. The *certain* dataset includes data after applying all filters. The *certain+NA* dataset includes also observations with unknown coordinates uncertainty (GBIF). Data downloaded in February 2019.

	**Global**			**European**		
Species	Occur.	Certain dataset	Certain+NA dataset	Occur.	Certain dataset	Certain+NA dataset
**Mammals**						
Cervus nippon	4601	397	457	3037	368	398
Myocastor coypus	29342	1521	2052	27333	1328	1382
Neovison vison	27616	2867	3151	26623	2444	2657
Nyctereutes procyonoides	9606	452	689	2629	422	610
Ondatra zibethicus	20132	1863	2323	18256	1223	1585
Procyon lotor	13394	2220	2637	3502	405	448
Rattus norvegicus	55552	3331	3882	53982	3064	3499
Sciurus carolinensis	120174	1943	2088	105242	631	633
Tamias sibiricus	1810	77	93	1736	71	72
**Birds**						
Branta canadensis	5541972	5779	22955	863923	3704	5849
Oxyura jamaicensis	903838	1185	13030	27115	528	2965
Psittacula krameri	165859	806	5745	73555	565	3028
Threskiornis aethiopicus	147726	530	2695	4597	281	328
**Amphibians**						
Lithobates catesbeianus	13029	1447	1941	3507	51	52
**Reptiles**						
Trachemys scripta	23561	2518	2867	12159	1212	1248

Table S1.6. Algorithms selected to fit models on individual species. Default specifications are shown in regular text (information provided by BIOMOD2 function *Print_Default_ModelingOptions()*); particular specifications are marked in bold italics.

Algorithm	Acronym	Specifications
Generalized linear model	GLM	type = 'quadratic', interaction.level = 0, myFormula = NULL, test = 'AIC', family = binomial(link = 'logit'), mustart = 0.5, control = glm.control(epsilon = 1e-08, maxit = 50, trace = FALSE)
Generalized additive model	GAM	algo = 'GAM_mgcv', type = 's_smoother', ***k = 3***, interaction.level = 0, myFormula = NULL, family = binomial(link = 'logit'), method = 'GCV.Cp', optimizer = c('outer','newton'), select = FALSE, knots = NULL, paraPen = NULL, control = list(nthreads = 1, irls.reg = 0, epsilon = 1e-07, maxit = 200, trace = FALSE, mgcv.tol = 1e-07, mgcv.half = 15, rank.tol = 1.49011611938477e-08 , nlm = list(ndigit=7, gradtol=1e-06, stepmax=2, steptol=1e-04, iterlim=200, check.analyticals=0), optim = list(factr=1e+07), newton = list(conv.tol=1e-06, maxNstep=5, maxSstep=2, maxHalf=30, use.svd=0), outerPIsteps = 0, idLinksBases = TRUE, scalePenalty = TRUE, efs.lspmax = 15, efs.tol = 0.1, keepData = FALSE, scale.est = fletcher, edge.correct = FALSE)
Flexible discriminant analysis	FDA	method = 'mars', add_args = NULL
Generalized boosting model	GBM	distribution = 'bernoulli', ***n.trees = 1000***, interaction.depth = 7, n.minobsinnode = 5, shrinkage = 0.001, bag.fraction = 0.5, train.fraction = 1, cv.folds = 3, keep.data = FALSE, verbose = FALSE, perf.method = 'cv'
Maximum entropy	MAXENT. Philips	***path_to_maxent.jar = 'MyMaxentlocation'***, memory_allocated = 512, background_data_dir = 'default', maximumbackground = 'default', maximumiterations = 200, visible = FALSE, linear = TRUE, quadratic = TRUE, product = TRUE, threshold = TRUE, hinge = TRUE, lq2lqptthreshold = 80, l2lqthreshold = 10, hingethreshold = 15, beta_threshold = -1, beta_categorical = -1, beta_lqp = -1, beta_hinge = -1, betamultiplier = 1, defaultprevalence = 0.5

SI 2: Complementary results

SI 2.1 Individual species models

a) Models included in the main text

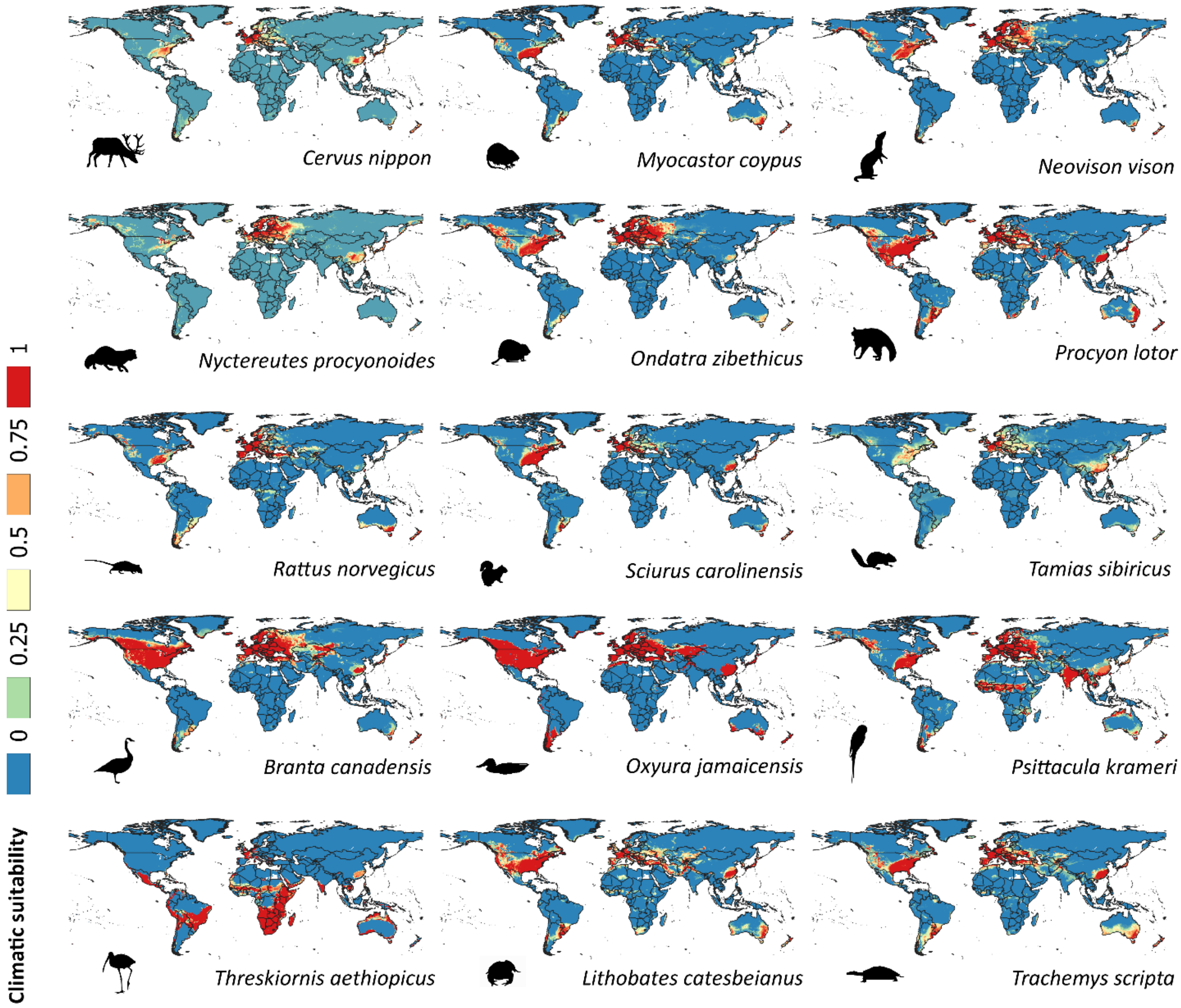

Figure S2.1.1. Climatic suitability predictions obtained from the global ensemble SDMs of each IATV species fitted with the *certain+NA* dataset (continuous values, ranging from 0 to 1). Blue colors show low-suitability areas. Red colors indicate high-suitability areas. This figure was generated with QGIS v.3.2.3[7] (www.qgis.org).

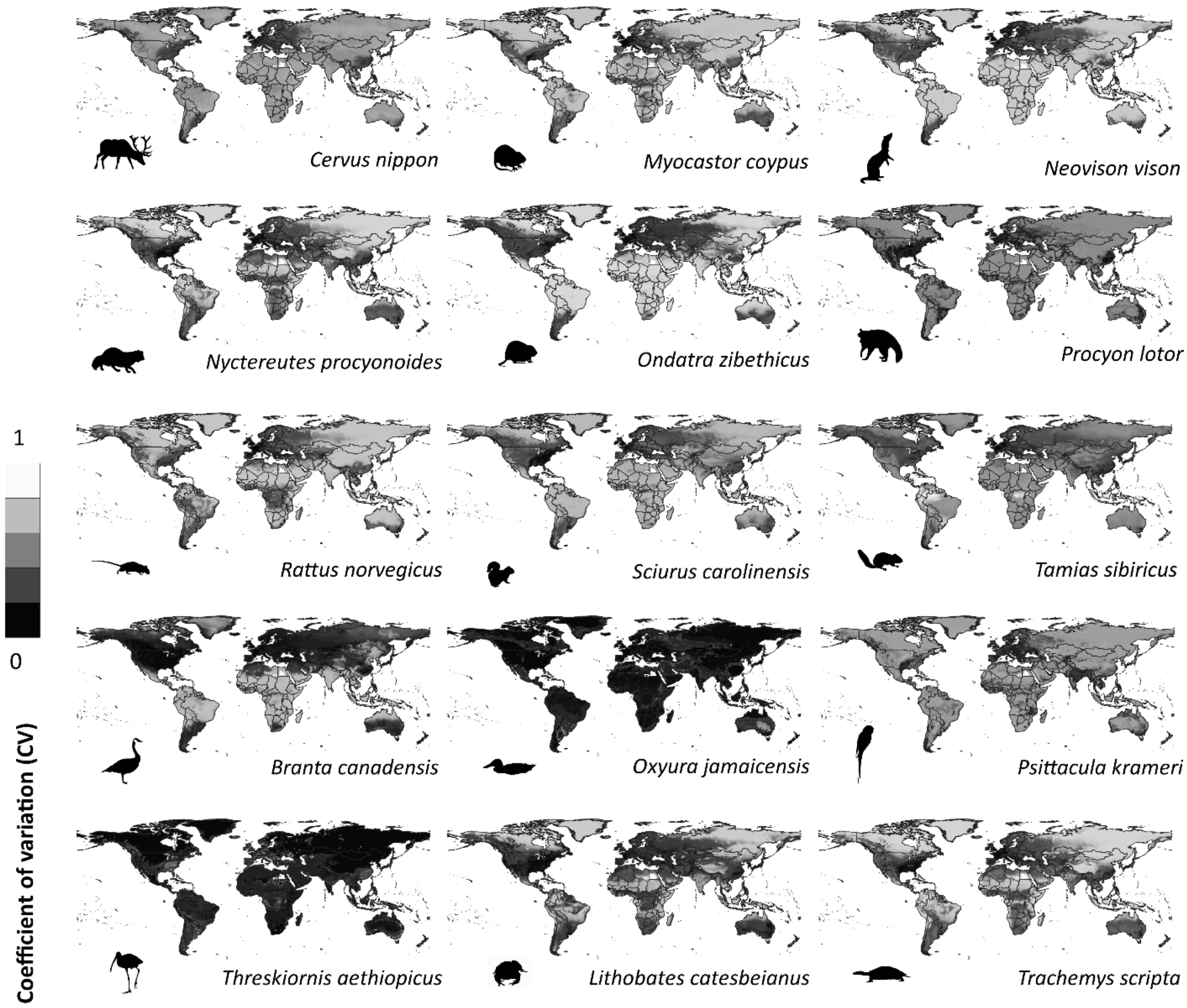

Figure S2.1.2. Coefficients of variation of the climatic suitability predictions obtained from the global ensemble SDMs for each of the IATV species fitted using the *certain+NA* dataset (normalized between 0 and 1). Darker colors represent low CV values, which indicates agreement among predictions. Lighter colors indicate high CV values of low agreement among predictions. This figure was generated with QGIS v.3.2.3[7] (www.qgis.org).

Table S2.1.1. Variable importance according to the European ensemble models for each species. Grey cells highlight the top-four variables for each species' ensemble model.

Species	Predictors: *Climate*						*Land use*							*Water availability*			*Topography*	*Accessibility*
	Temp.seasonality	Mean temp. wettest	Isothermality	Prec. seasonality	Prec. warmest	Prec. coldest	Forested primary	Non-forested	Potentially non-	Urban	Managed pasture	Rangeland	Croplands	River density	Water bodies	Distance to the coast	Roughness	Travel time to major cities
**Mammals**																		
Cervus nippon	0.60	0.01	0.04	0.15	0.07	0.01	0.09	0.09	0.00	0.01	0.05	0.07	0.01	0.00	0.00	0.02	0.03	0.04
Myocastor coypus	0.21	0.05	0.05	0.19	0.09	0.17	0.06	0.04	0.01	0.01	0.02	0.01	0.03	0.01	0.01	0.03	0.03	0.06
Neovison vison	0.20	0.16	0.08	0.33	0.08	0.11	0.02	0.00	0.03	0.02	0.03	0.00	0.06	0.00	0.02	0.03	0.03	0.08
Nyctereutes procyonoides	0.21	0.07	0.32	0.21	0.11	0.19	0.04	0.01	0.00	0.01	0.04	0.01	0.05	0.00	0.01	0.02	0.17	0.07
Ondatra zibethicus	0.07	0.05	0.05	0.27	0.14	0.04	0.03	0.01	0.01	0.01	0.02	0.02	0.01	0.00	0.01	0.02	0.03	0.07
Procyon lotor	0.37	0.01	0.10	0.29	0.16	0.03	0.07	0.01	0.01	0.02	0.01	0.01	0.02	0.00	0.01	0.10	0.04	0.02
Rattus norvegicus	0.54	0.07	0.09	0.07	0.11	0.13	0.02	0.01	0.01	0.03	0.02	0.00	0.02	0.00	0.00	0.01	0.00	0.05
Sciurus carolinensis	0.49	0.01	0.07	0.15	0.04	0.05	0.07	0.10	0.02	0.01	0.06	0.01	0.01	0.01	0.01	0.05	0.02	0.09
Tamias sibiricus	0.24	0.01	0.06	0.44	0.08	0.04	0.03	0.07	0.05	0.25	0.05	0.03	0.03	0.02	0.01	0.03	0.05	0.10
**Birds**																		
Branta canadensis	0.11	0.07	0.07	0.11	0.51	0.10	0.03	0.00	0.01	0.03	0.09	0.02	0.01	0.00	0.06	0.04	0.04	0.09
Oxyura jamaicensis	0.37	0.07	0.16	0.25	0.09	0.02	0.01	0.01	0.03	0.05	0.02	0.02	0.05	0.01	0.03	0.05	0.03	0.02
Psittacula krameri	0.52	0.04	0.09	0.04	0.05	0.04	0.07	0.00	0.02	0.16	0.02	0.01	0.02	0.01	0.00	0.03	0.02	0.15
Threskiornis aethiopicus	0.36	0.05	0.04	0.04	0.04	0.19	0.05	0.01	0.01	0.05	0.04	0.05	0.06	0.04	0.03	0.05	0.25	0.09
**Amphibians**																		
Lithobates catesbeianus	0.19	0.04	0.44	0.10	0.10	0.23	0.02	0.07	0.16	0.20	0.02	0.03	0.05	0.01	0.04	0.07	0.26	0.02
**Reptiles**																		
Trachemys scripta	0.22	0.06	0.21	0.08	0.04	0.08	0.05	0.01	0.00	0.04	0.01	0.01	0.01	0.01	0.00	0.01	0.02	0.16

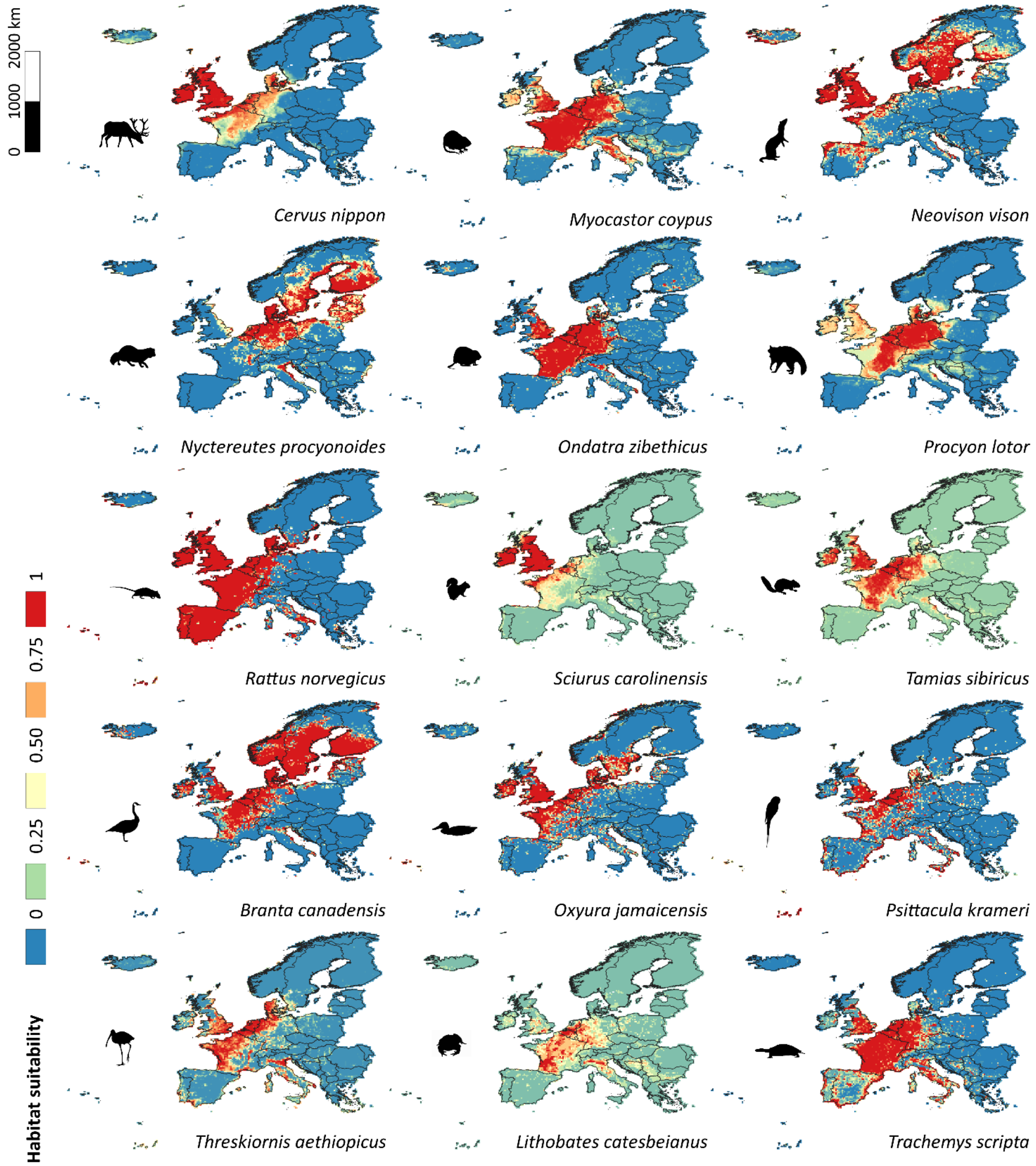

Figure S2.1.3. Environmental suitability predictions obtained from the European ensemble SDMs of each IATV species fitted with the *certain* dataset and after fitting the global model also using the *certain+NA* dataset (continuous values, ranging from 0 to 1). Blue colors show low-suitability areas, red colors indicate high-suitability areas. This figure was generated with QGIS v.3.2.3[7] (www.qgis.org).

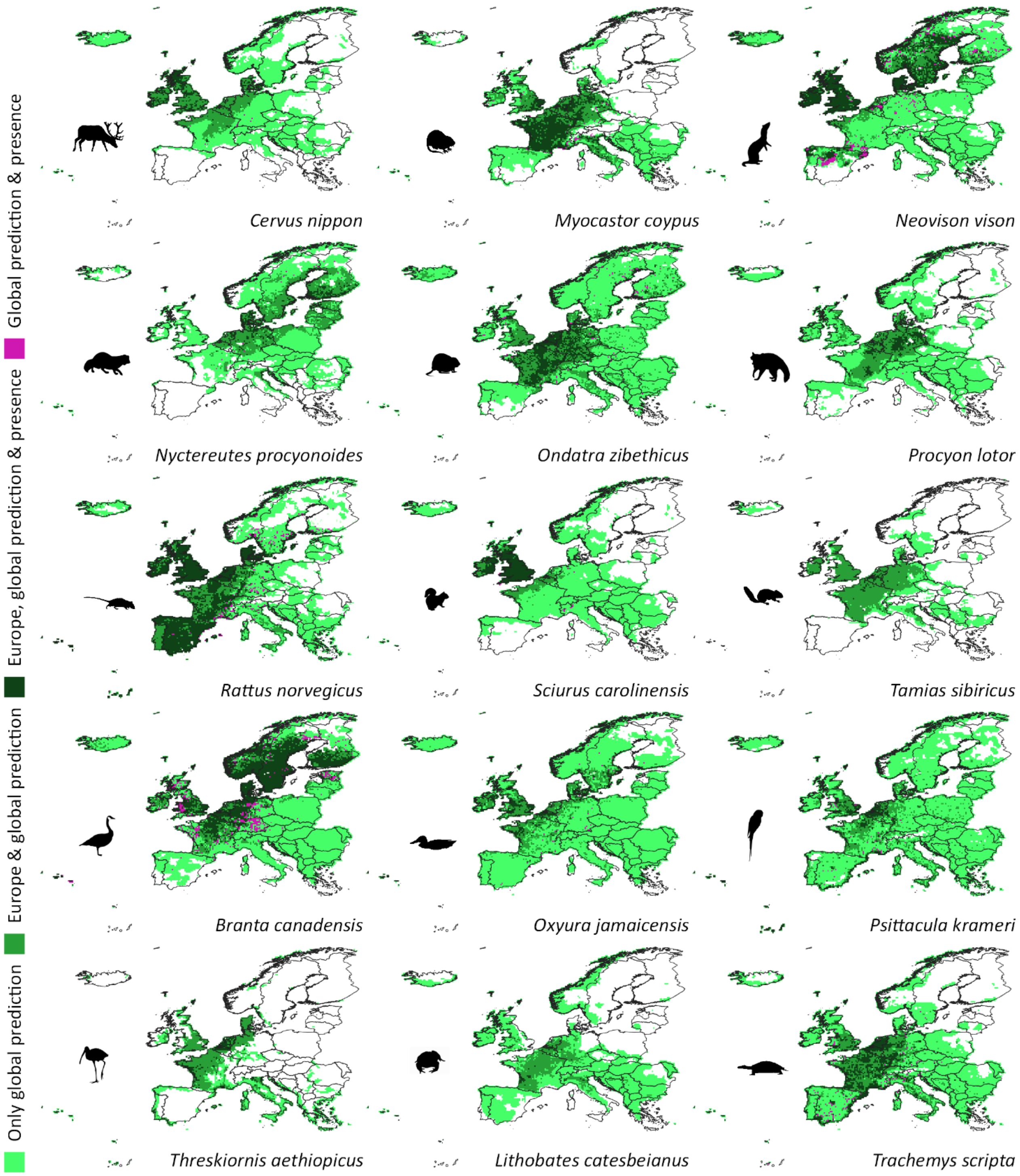

Figure S2.1.4. Overlapped results of the binary predictions (global and European) and the presence data. Light green color represents areas where the species presence is predicted only by the global model. Medium green depicts areas where both the global and the European models predict the potential presence of a species as shown by binary maps. Dark green color shows areas where both the global and the European models predict the potential presence of a species, and the species presence is reported (*certain* dataset) occur. Pink areas shows grid-cells where the global model predict the potential presence of a species as for the binary prediction and the species presence is reported (*certain* dataset). This figure was generated with QGIS v.3.2.3[7] (www.qgis.org).

b) Additional models considering different datasets of IATV presences

We used three different combinations of datasets to fit our models at the European scale. The first dataset combination is the one described in the main text of the manuscript and consisted of using the *certain+NA* dataset to fit the global model and the *certain* dataset to fit the European model, respectively. Additionally, we fitted both models, the global and European, using the *certain* datasets (b.1), and then again both models using the *certain+NA* datasets (b.2; Table S1.5 summarizes sample sizes for each case). We compared the performance of the models using sensitivity, specificity and TSS values, and we spatially compared the continuous outputs calculating the pixel-based Bhattacharyya distance (BD; Huang et al. 2018).

b.1) Using the *certain* dataset in the global and European models

Using only *certain* presences in the global models (Fig. S2.1.5) resulted in a slightly higher predictive ability attending to TSS, sensitivity and specificity (Table S2.1.2 *vs.* Table 3). Besides, this type of model showed a lower variability in the predictions across the ensemble models for 8 of the 15 species, particularly for mammals (CV_{range} = 0.13-0.70, Table S2.1.2 and Fig. S2.1.6). The dissimilarity between predictions was higher for bird species (~0.1 Bhattacharyya Distance; Table S2.1.2) and most pronounced in North America, Central Asia, Eastern Europe and the sub-Saharian belt (Fig. S2.1.7).

The European models whose pseudo-absences were weighted based on global models fitted with *certain* datasets (Fig. S2.1.8) were similarly accurate than those based on global models fitted with *certain+NA* datasets (Table S2.1.3 *vs.* Table 2). Five species showed lower TSS values (max. ΔTSS 0.19) and four species showed higher values (max. ΔTSS 0.17); the remaining six species presented similar values. Sensitivity and specificity increased in half of the cases. The uncertainty among predictions was also variable among species. The mean CV of predictions was higher in seven of the 15 species, lower in five species, and nearly equal for the three remaining species (Table S2.1.3). The proportion of range filling also varied among species, with seven and eight species showing higher and lower values, respectively (Table S2.1.3 *vs*. Table 2). The spatial dissimilarity between predictions of the European models derived from the global models using *certain* datasets, and the European models derived from the global model fitted with *certain+NA* datasets, was notably lower than when comparing global models. *Lithobates catesbeianus* exhibited the maximum value of dissimilarity (BD = 0.079; Table S2.1.3; Fig. S2.1.9).

Table S2.1.2. Predictive accuracy of the global models using the *certain* dataset. n indicates the number of presence points used to fit the model. TSS is the true skill statistic. Sensitivity is the proportion of presences correctly predicted. Specificity is the proportion of absences correctly predicted. Cut-off shows the value of suitability (0-1) that minimizes TSS, and it was used to convert from continuous to binary ensemble prediction. Mean CV is the mean CV over all grid-cells of the ensemble prediction. Dissimilarity is calculated as the Bhattacharyya distance ranging from 0 (identical predictions) to 1 (highly different predictions) and averaged over all grid-cells for each species.

Species	n	TSS	Sensitivity	Specificity	Cut-off	Mean CV	Dissimilarity
**Mammals**							
Cervus nippon	397	0.92	96.97	95.07	0.46	0.66	0.003
Myocastor coypus	2867	0.84	95.09	88.39	0.49	0.62	0.080
Neovison vison	1521	0.90	95.25	94.51	0.59	0.59	0.001
Nyctereutes procyonoides	452	0.91	97.56	93.89	0.56	0.54	0.009
Ondatra zibethicus	1861	0.77	94.82	82.24	0.21	0.49	0.005
Procyon lotor	2218	0.75	90.21	84.77	0.70	0.13	0.014
Rattus norvegicus	3329	0.87	93.57	93.73	0.61	0.59	0.009
Sciurus carolinensis	1942	0.86	95.71	90.65	0.34	0.54	0.001
Tamias sibiricus	77	0.89	92.21	96.63	0.40	0.70	0.017
**Birds**							
Branta canadensis	5777	0.79	94.07	84.91	0.27	0.50	0.081
Oxyura jamaicensis	1184	0.74	93.55	80.45	0.41	0.41	0.107
Psittacula krameri	805	0.77	94.13	83.31	0.39	0.53	0.101
Threskiornis aethiopicus	530	0.81	94.89	86.49	0.48	0.65	0.116
**Amphibians**							
Lithobates catesbeianus	1441	0.80	91.05	89.03	0.58	0.58	0.011
**Reptiles**							
Trachemys scripta	2515	0.81	93.39	87.69	0.54	0.52	0.002

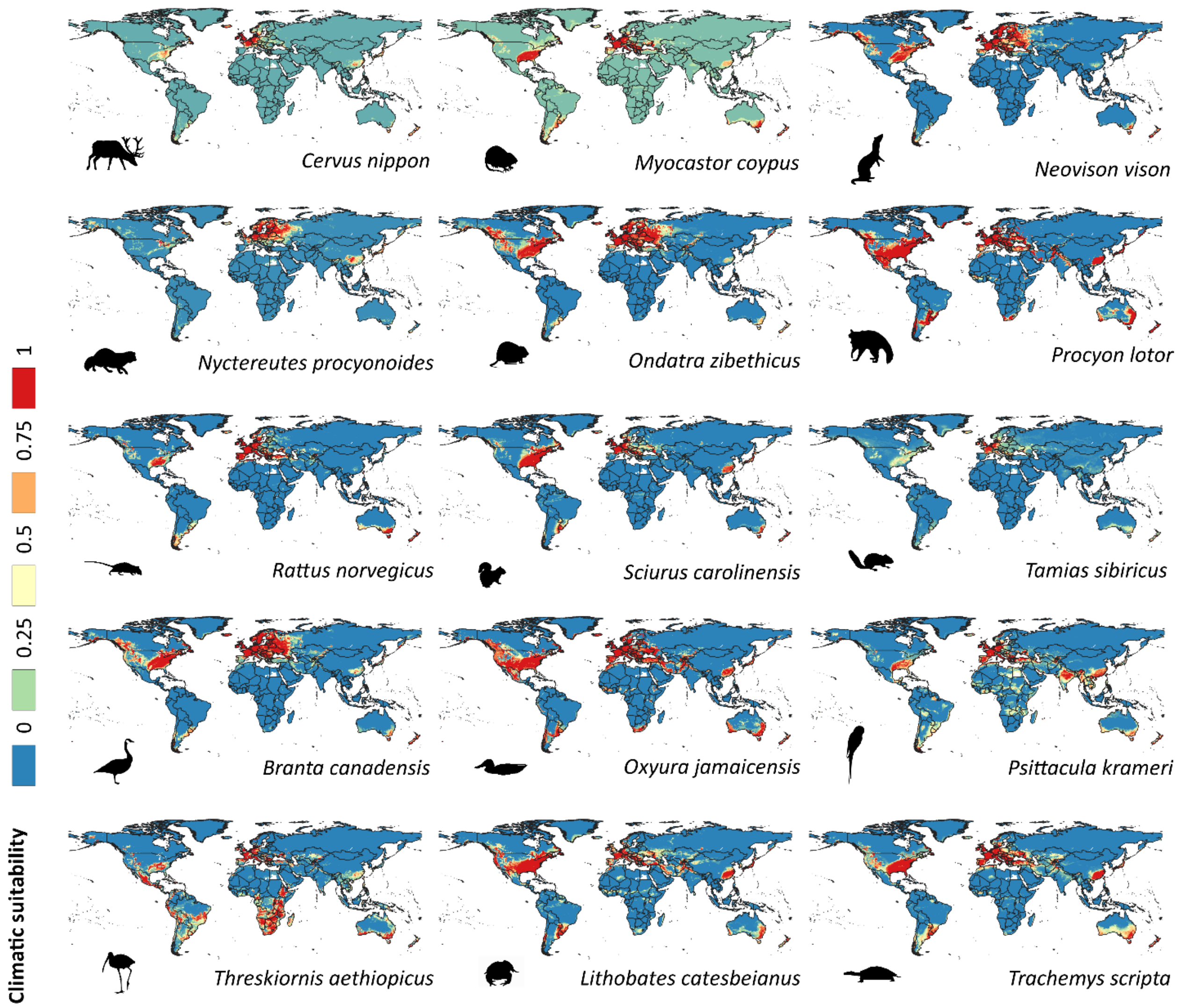

Figure S2.1.5. Climatic suitability predictions obtained from the global ensemble SDMs of each IATV species fitted with the *certain* dataset (continuous values, ranging from 0 to 1). Blue colors show low-suitability areas. Red colors indicate high-suitability areas. This figure was generated with QGIS v.3.2.3[7] (www.qgis.org).

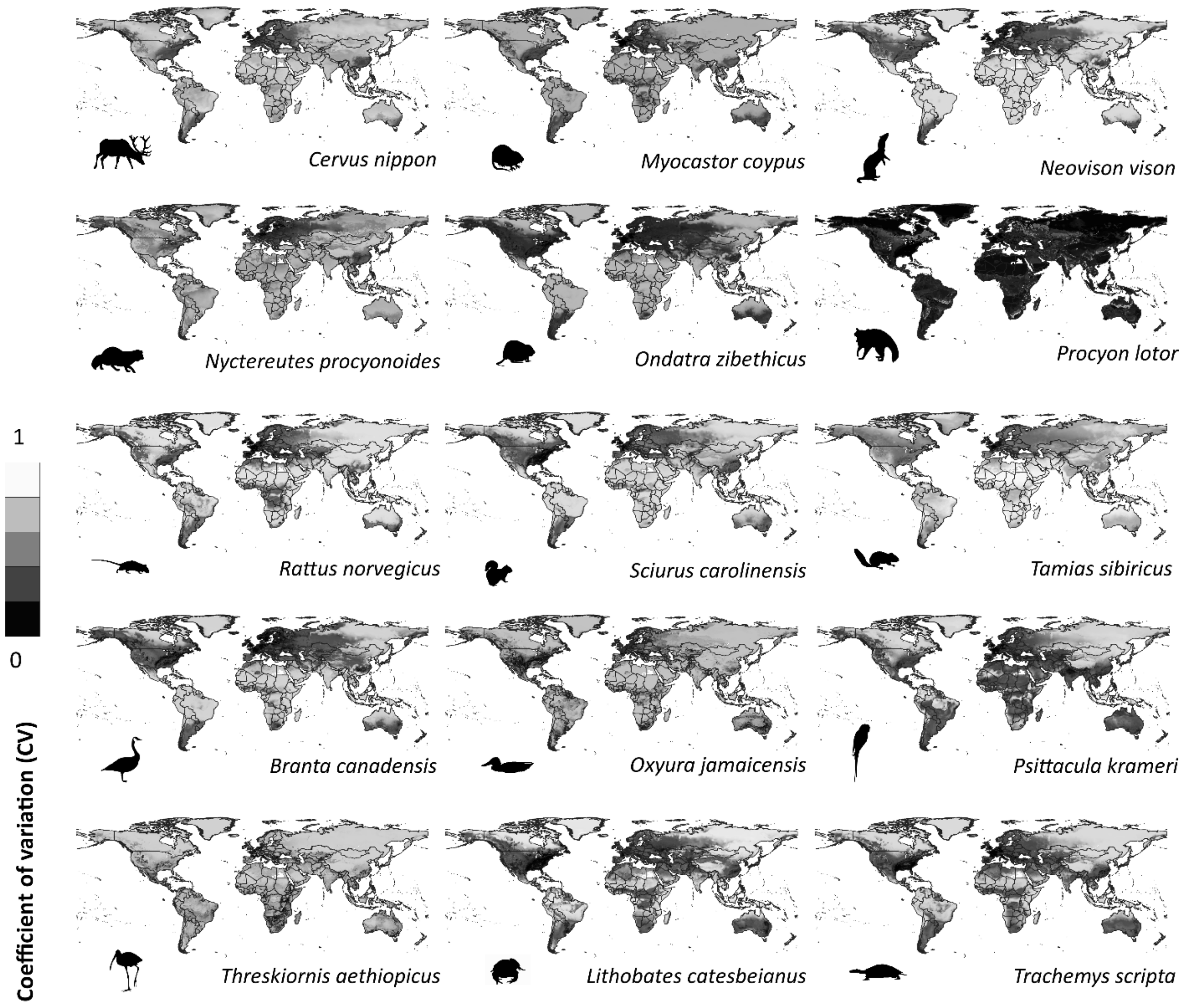

Figure S2.1.6. Coefficients of variation of the climatic suitability predictions obtained from the global ensemble SDMs of each IATV species fitted with the *certain* dataset (normalized between 0 and 1). Dark colors represent low CV values, meaning agreement between predictions. Light colors indicate high CV values or low agreement between predictions. This figure was generated with QGIS v.3.2.3[7] (www.qgis.org).

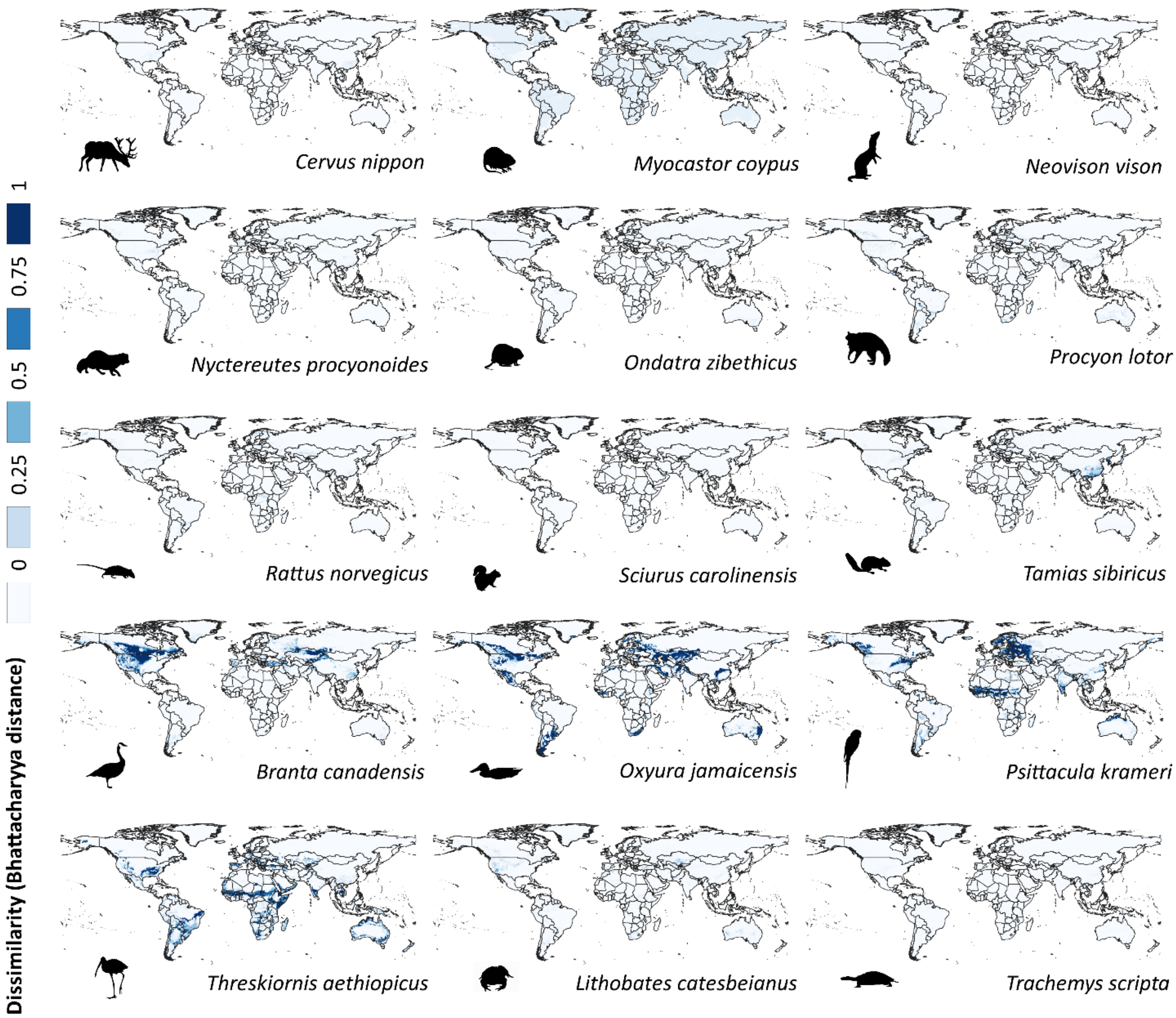

Figure S2.1.7. Measure of dissimilarity per grid-cell (Bhattacharyya distance) between global predictions based on the *certain+NA* (main text; Fig. S2.1.1) and the *certain* (Fig. S2.1.5) datasets. Darker color indicates larger differences between predictions. This figure was generated with QGIS v.3.2.3[7] (www.qgis.org).

Table S2.1.3. Predictive accuracy of the European models using the *certain* dataset to fit the global and European models. n indicates the number of presence points used to fit the model. TSS is the true skill statistic. Sensitivity is the proportion of presences correctly predicted. Specificity is the proportion of absences correctly predicted. Cut-off shows the value of suitability (0-1) that minimizes TSS, and it was used to convert from continuous to binary ensemble prediction. Mean CV is the mean CV over all grid-cells of the ensemble prediction. Range filling represents the fraction of the grid-cells classified as 'presence' in the binary map that overlapped with the observed records of species presences. Dissimilarity is calculated as the Bhattacharyya distance ranging from 0 (identical predictions) to 1 (highly different predictions) and averaged over all grid-cells for each species.

Species	n	TSS	Sensitivity	Specificity	Cut-off	Mean CV	Range filling	Dissimilarity
**Mammals**								
Cervus nippon	368	0.83	95.91	86.96	0.67	0.27	0.18	0.009
Myocastor coypus	1328	0.62	82.53	79.12	0.50	0.53	0.42	0.009
Neovison vison	2444	0.80	94.94	85.32	0.51	0.50	0.48	0.022
Nyctereutes procyonoides	422	0.73	87.83	84.83	0.75	0.56	0.16	0.023
Ondatra zibethicus	1223	0.67	80.76	86.06	0.30	0.15	0.38	0.015
Procyon lotor	405	0.85	95.80	88.76	0.57	0.34	0.22	0.007
Rattus norvegicus	3064	0.74	89.67	84.12	0.70	0.09	0.64	0.008
Sciurus carolinensis	631	0.93	95.34	97.50	0.63	0.36	0.56	0.001
Tamias sibiricus	71	0.86	98.59	87.71	0.42	0.27	0.04	0.012
**Birds**								
Branta canadensis	3704	0.67	84.08	82.90	0.58	0.62	0.66	0.018
Oxyura jamaicensis	528	0.73	90.08	82.72	0.58	0.41	0.18	0.019
Psittacula krameri	564	0.74	89.53	84.86	0.41	0.46	0.21	0.022
Threskiornis aethiopicus	281	0.78	90.32	88.02	0.74	0.28	0.14	0.013
**Amphibians**								
Lithobates catesbeianus	51	0.85	96.08	88.88	0.58	0.81	0.03	0.079
**Reptiles**								
Trachemys scripta	1210	0.72	86.20	85.69	0.58	0.17	0.39	0.017

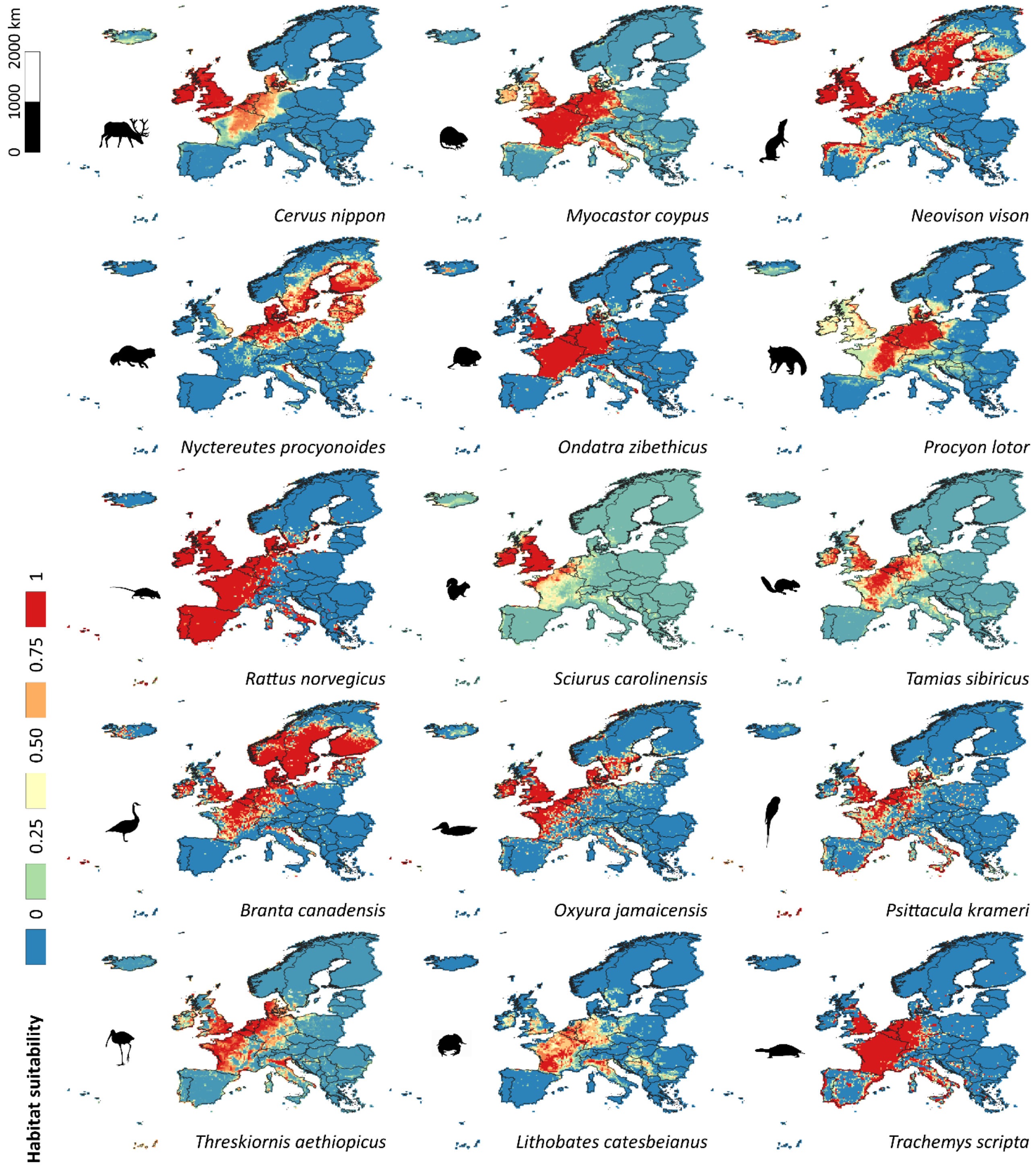

Figure S2.1.8. Environmental suitability predictions obtained from the European ensemble SDMs of each IATV species fitted with the *certain* dataset and after fitting the global model also using the *certain* dataset (continuous values, ranging from 0 to 1). Blue colors show low-suitability areas, red colors indicate high-suitability areas. This figure was generated with QGIS v.3.2.3[7] (www.qgis.org).

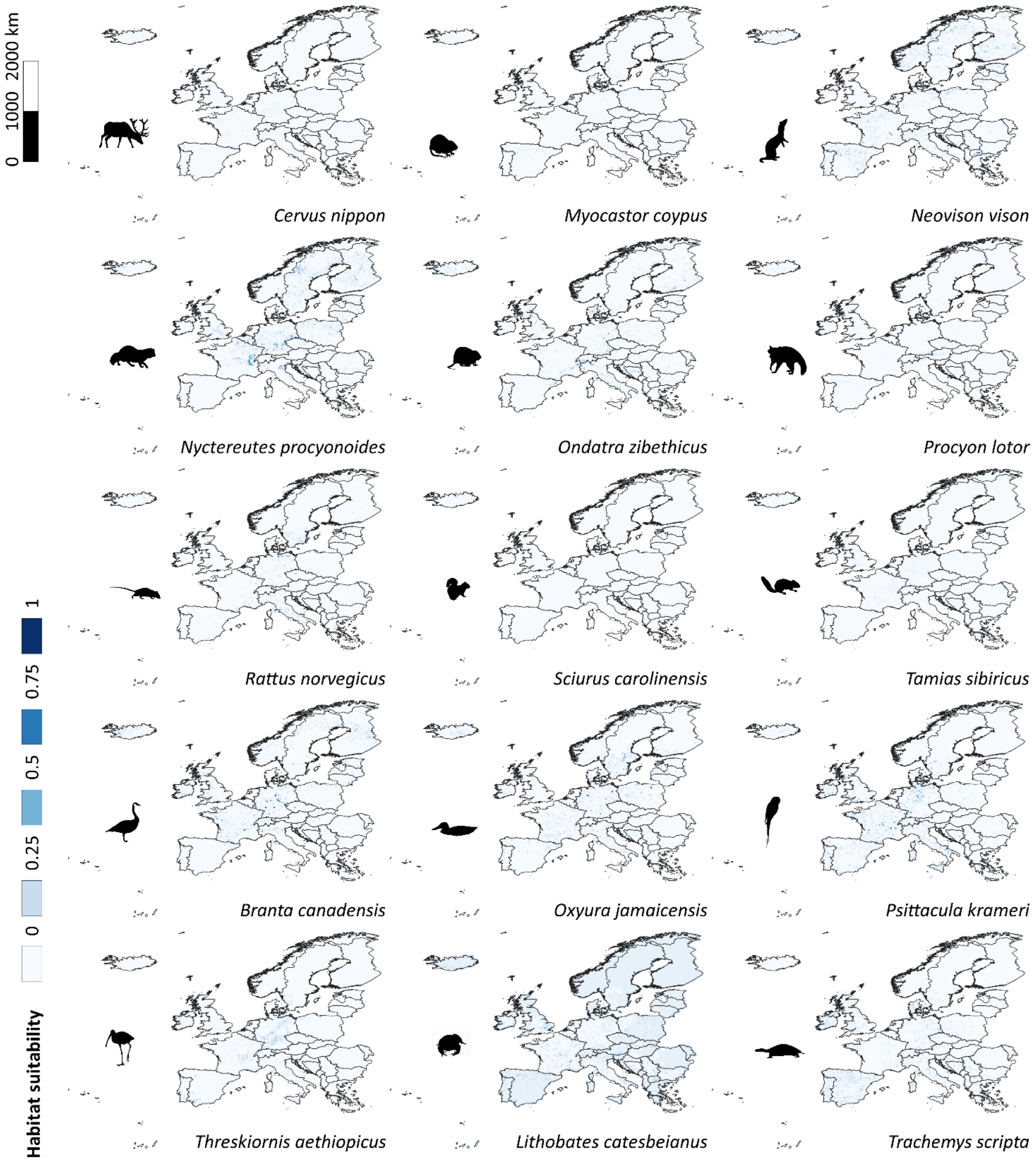

Figure S2.1.9. Measures of dissimilarity per grid-cells (Bhattacharyya distance) between European predictions based on the global model using the *certain+NA* (main text; Fig. S2.1.3) and the *certain* (Fig. S2.1.8) datasets. Darker color indicates larger differences between predictions. This figure was generated with QGIS v.3.2.3[7] (www.qgis.org).

b.2) Using the *certain+NA* datasets in the global and European models

In general, these models performed poorer than the models described in the main text of the article in terms of TSS and sensitivity, although for some species, the specificity (i.e. correct prediction of absences) increased. Conversely, the variability within ensemble models was generally higher as indicated by CV values (Table S2.1.4 *vs*. Table 2). The proportion of range filling was predicted to be higher in this version of the models than in the models shown in the main text, except for the cases of *C.nippon, L.catesbeianus* and *T.scripta* (Table S2.1.4 vs. Table 2). Mean values of spatial dissimilarity (Bhattacharyya distance) per species, relative to the main-text model, were generally higher than in the case of the model fitted with both *certain* datasets, with particularly large differences in the cases of *P.krameri* and *O.jamaicensis* (Fig. S2.1.11).

Table S2.1.4. Predictive accuracy of the European models using the *certain+NA* dataset to fit the global and European models. n indicates the number of presence points used to fit the model. TSS is the true skill statistic. Sensitivity is the proportion of presences correctly predicted. Specificity is the proportion of absences correctly predicted. Cut-off shows the value of suitability (0-1) that minimizes TSS, and it was used to convert from continuous to binary ensemble prediction. Mean CV is the mean CV over all grid-cells of the ensemble prediction. Range filling represents the fraction of the grid-cells classified as 'presence' in the binary map that overlapped with the observed records of species presences. Dissimilarity is calculated as the Bhattacharyya distance ranging from 0 (identical predictions) to 1 (highly different predictions) and averaged over all grid-cells for each species.

Species	n	TSS	Sensitivity	Specificity	Cut-off	Mean CV	Range filling	Dissimilarity
**Mammals**								
Cervus nippon	398	0.80	95.97	84.45	0.57	0.31	0.17	0.006
Myocastor coypus	1382	0.80	90.27	89.89	0.74	0.44	0.51	0.005
Neovison vison	2657	0.62	80.50	82.04	0.70	0.60	0.53	0.044
Nyctereutes procyonoides	610	0.67	76.04	90.73	0.91	0.36	0.29	0.044
Ondatra zibethicus	1585	0.68	80.73	87.55	0.58	0.21	0.48	0.041
Procyon lotor	448	0.83	93.75	88.82	0.64	0.38	0.24	0.005
Rattus norvegicus	3499	0.71	86.73	84.68	0.58	0.09	0.68	0.029
Sciurus carolinensis	633	0.92	95.52	96.96	0.58	0.37	0.55	0.004
Tamias sibiricus	72	0.86	95.83	90.39	0.60	0.22	0.04	0.002
**Birds**								
Branta canadensis	5849	0.50	66.00	83.58	0.75	0.58	0.75	0.061
Oxyura jamaicensis	2965	0.21	56.37	64.60	0.91	0.30	0.30	0.346
Psittacula krameri	3028	0.23	51.42	71.58	0.90	0.33	0.33	0.293
Threskiornis aethiopicus	328	0.74	86.11	88.31	0.75	0.30	0.15	0.007
**Amphibians**								
Lithobates catesbeianus	52	0.83	98.08	85.22	0.30	0.80	0.03	0.080
**Reptiles**								
Trachemys scripta	1248	0.73	89.83	83.30	0.41	0.46	0.37	0.013

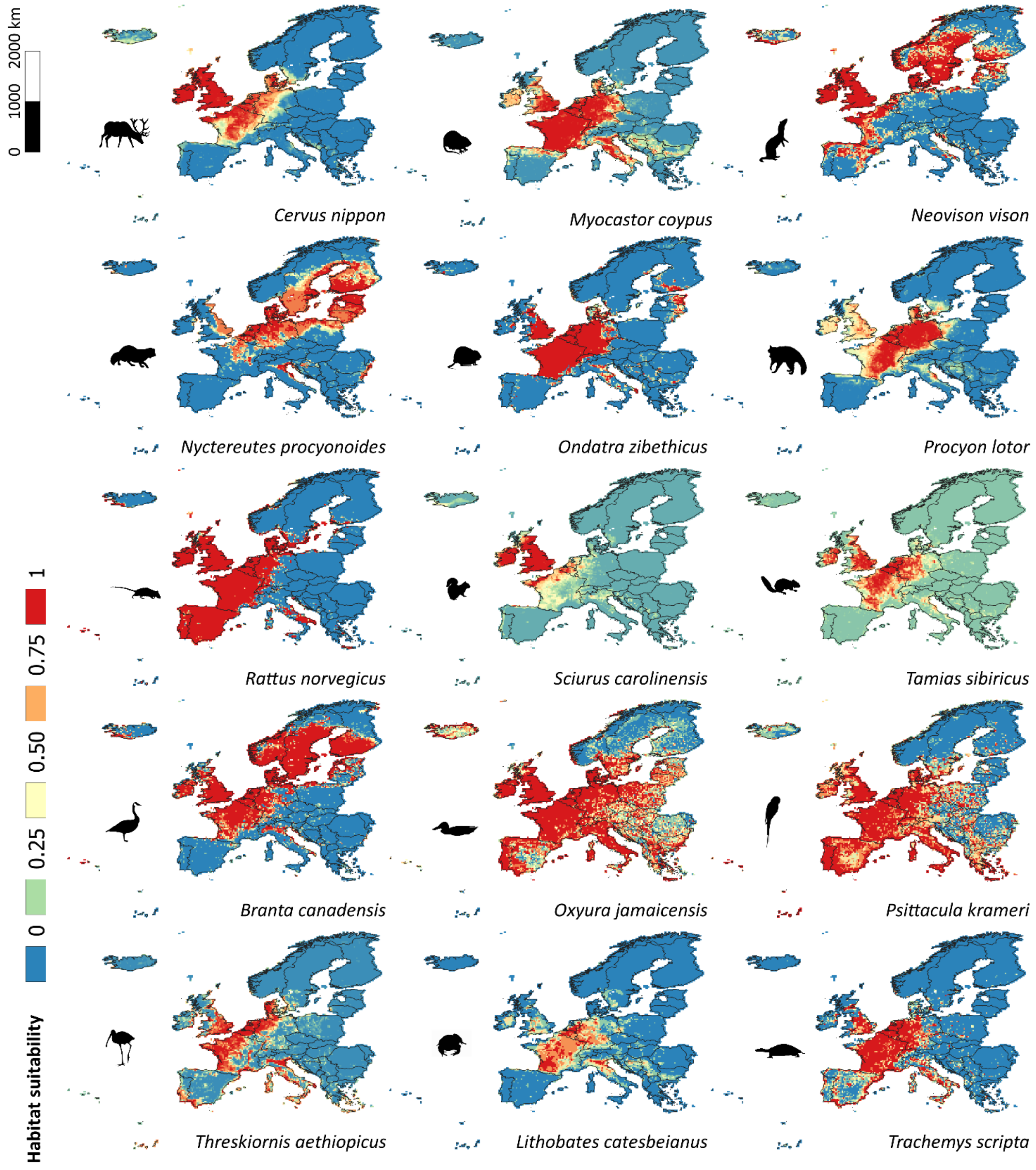

Figure S2.1.10. Environmental suitability predictions obtained from the European ensemble SDMs for each IATV species fitted with the *certain+NA* dataset and after fitting the global model also using the *certain+NA* dataset (continuous values, ranging from 0 to 1). Blue colors show low-suitability areas, red colors indicate high-suitability areas. This figure was generated with QGIS v.3.2.3[7] (www.qgis.org).

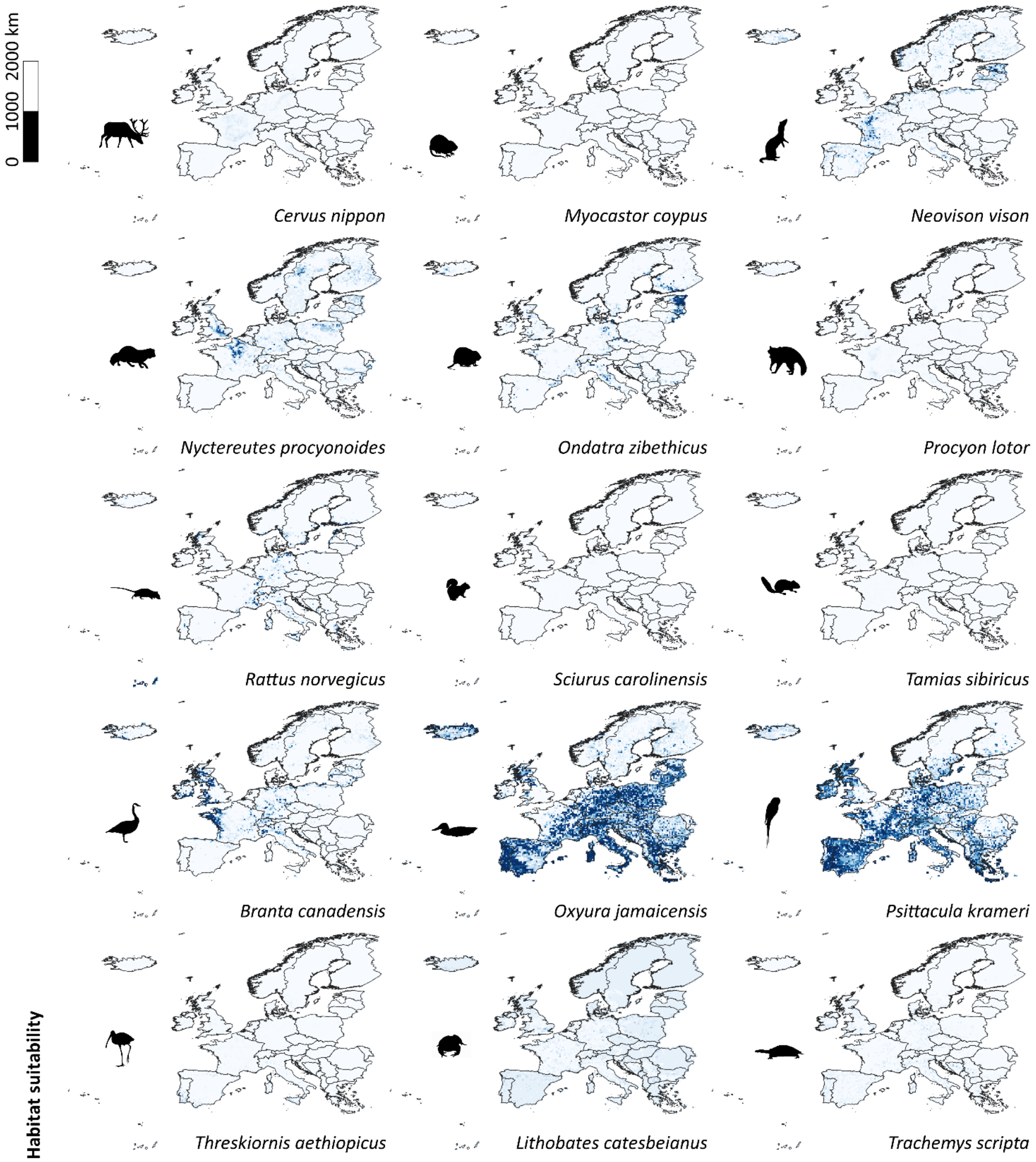

Figure S2.1.11. Measures of dissimilarity per grid-cells (Bhattacharyya distance) between European predictions using the *certain* (main text; Fig. S2.1.3) and the *certain+NA* (Fig. S2.1.10) datasets. Both models are based on global models using *certain+NA* datasets. Darker pixels indicate larger differences between predictions. This figure was generated with QGIS v.3.2.3[7] (www.qgis.org).

SI 2.2 Multi-species summary

a) Models included in the main text

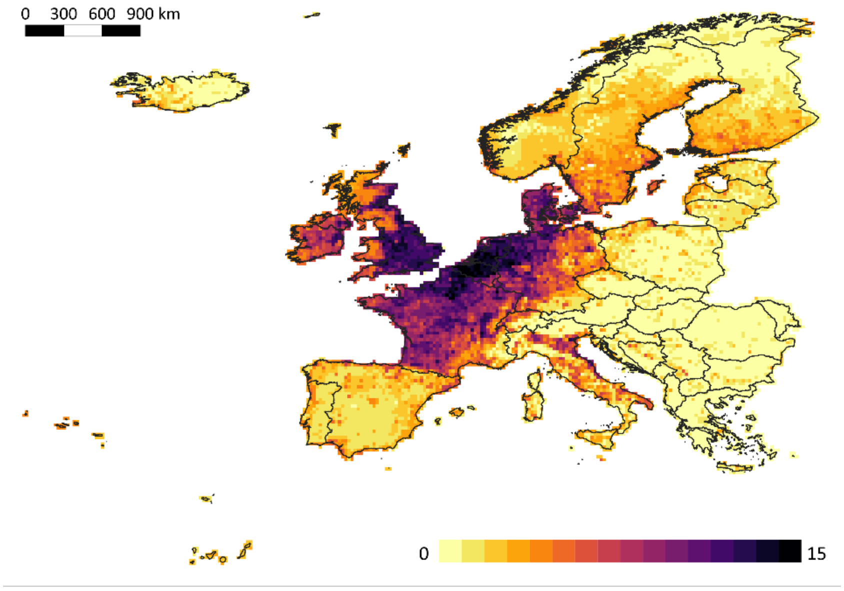

Figure S2.2.1. Predicted richness of invasive alien terrestrial vertebrate (IATV) derived from the European models fitted with the *certain* dataset, and after fitting the global model using the *certain+NA* dataset. Units indicate the number of species per grid-cell scored as 'presence' in the binary map of predictions. This figure was generated with QGIS v.3.2.3[7] (www.qgis.org).

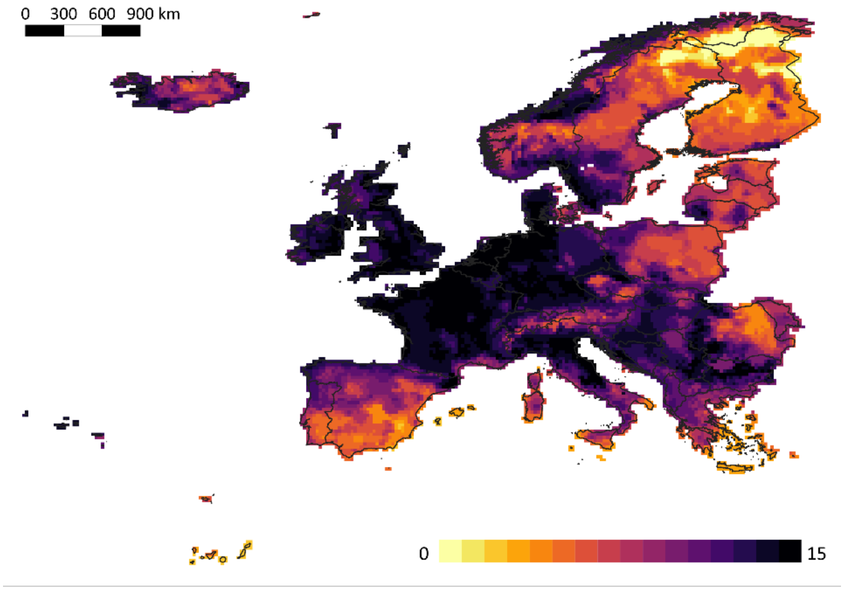

Figure S2.2.2. Predicted richness of invasive alien terrestrial vertebrate (IATV) derived from the global models using the *certain+NA* dataset. Units indicate the number of species per grid-cell scored as 'presence' in the binary map of predictions. This figure was generated with QGIS v.3.2.3[7] (www.qgis.org).

b) Additional models considering different datasets of IATV presences

b.1) Using the *certain* datasets in the global and European models

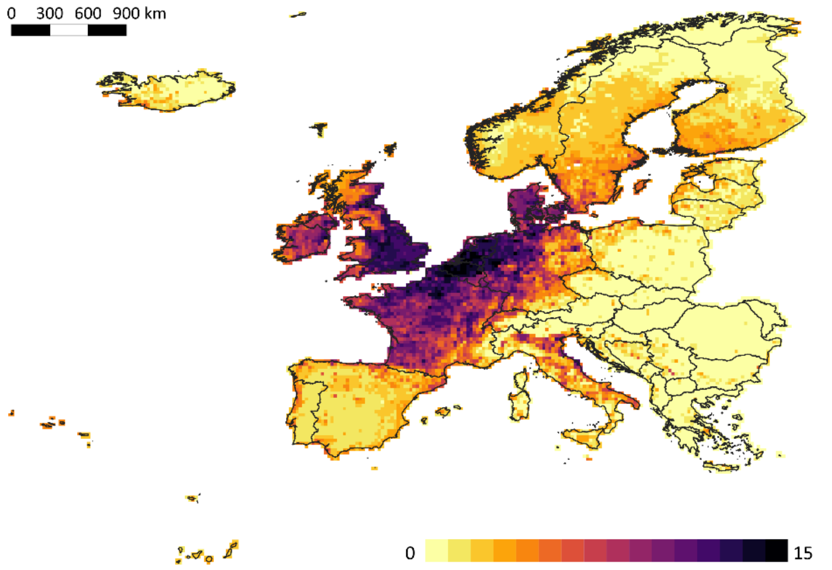

Figure S2.2.3. Predicted richness of invasive alien terrestrial vertebrates (IATV) derived from the European models fitted with the *certain* dataset, and after fitting the global model also using the *certain* dataset. Units indicate the number of species per grid-cell scored as 'presence' in the binary map of predictions. This figure was generated with QGIS v.3.2.3[7] (www.qgis.org).

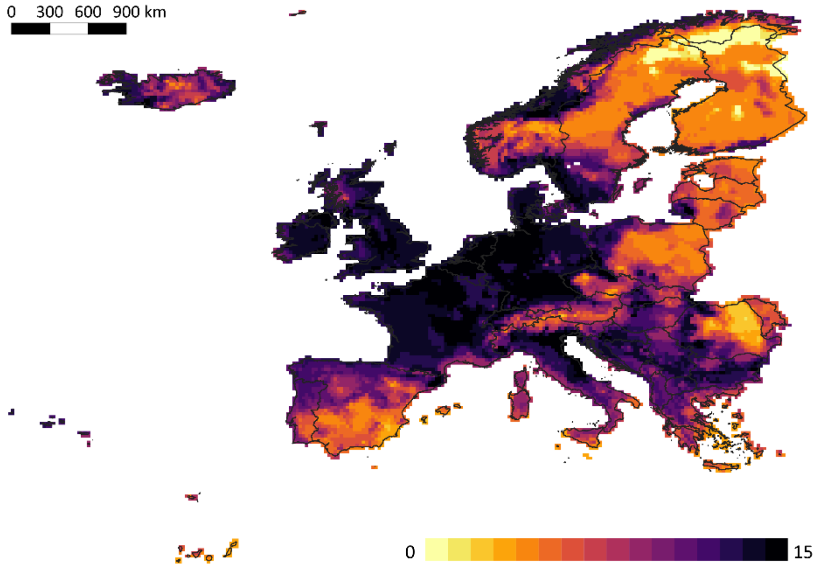

Figure S2.2.4. Predicted richness of invasive alien terrestrial vertebrates (IATV) derived from the global models using the *certain* dataset. Units indicate the number of species per grid-cell scored as 'presence' in the binary map of predictions. This figure was generated with QGIS v.3.2.3[7] (www.qgis.org).

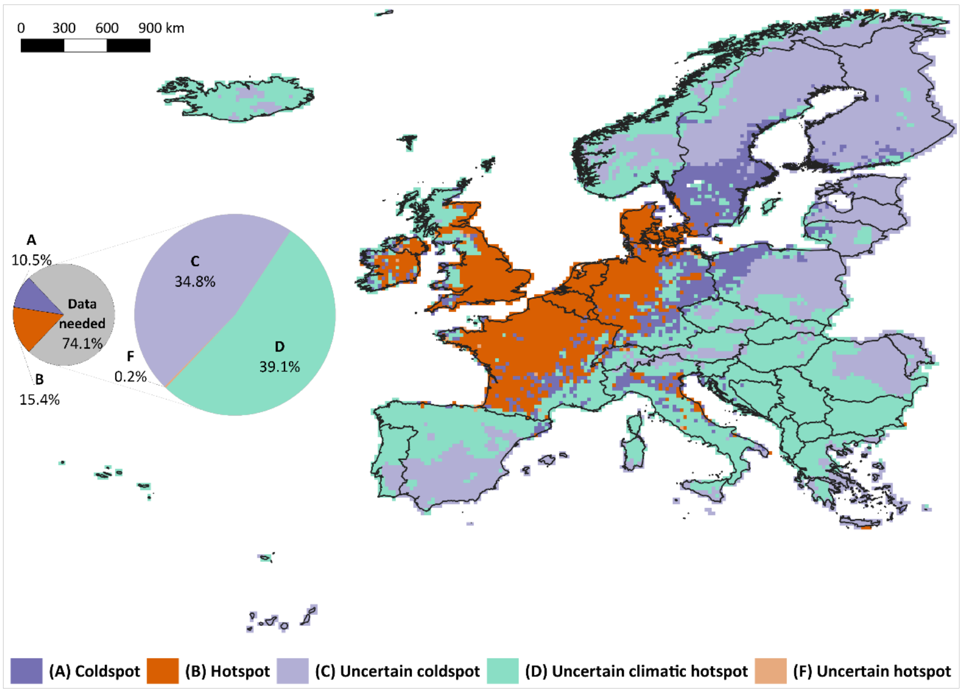

Figure S2.2.5. Priority management areas obtained from the application of the classification criteria described in Fig.1 (main text). The pie-chart to the left represents the proportion of grid-cells that belong to each class after aggregating all the categories under the 'data-needed' group, i.e. zones C to F. The pie-chart to the right represents the percentage of grid-cells within each zone in the 'data-needed' group, i.e. zones C to F (grey area). No grid-cell was classified as E (uncertain environmental hotspot) according to our criteria. *Certain* datasets were used to fit global and European models. This figure was generated with QGIS v.3.2.3[7] (www.qgis.org).

b.2) Using the *certain+NA* datasets in the global and European models

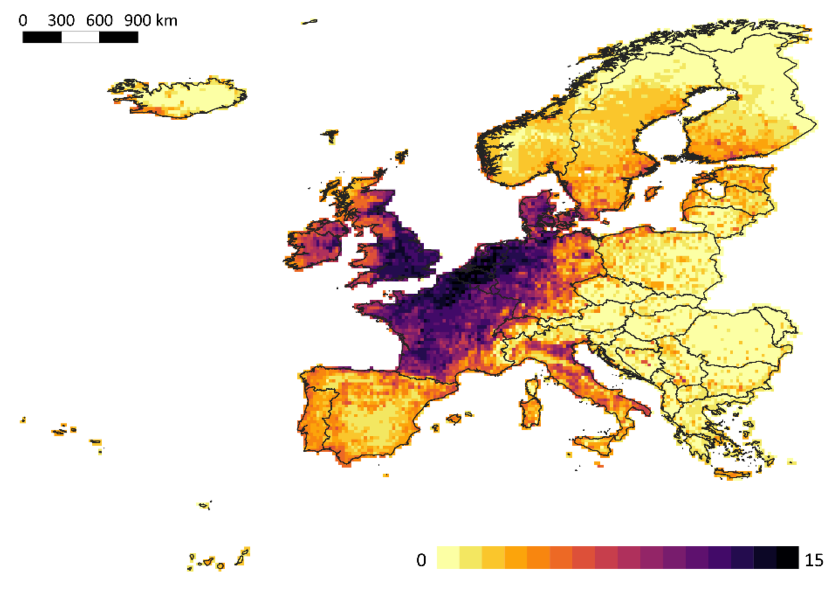

Figure S2.2.6. Predicted richness of invasive alien terrestrial vertebrates (IATV) derived from the European models fitted with the *certain+NA* dataset, and after fitting the global model also using the *certain+NA* dataset. Units indicate the number of species per grid-cell scored as 'presence' in the binary map of predictions. This figure was generated with QGIS v.3.2.3[7] (www.qgis.org).

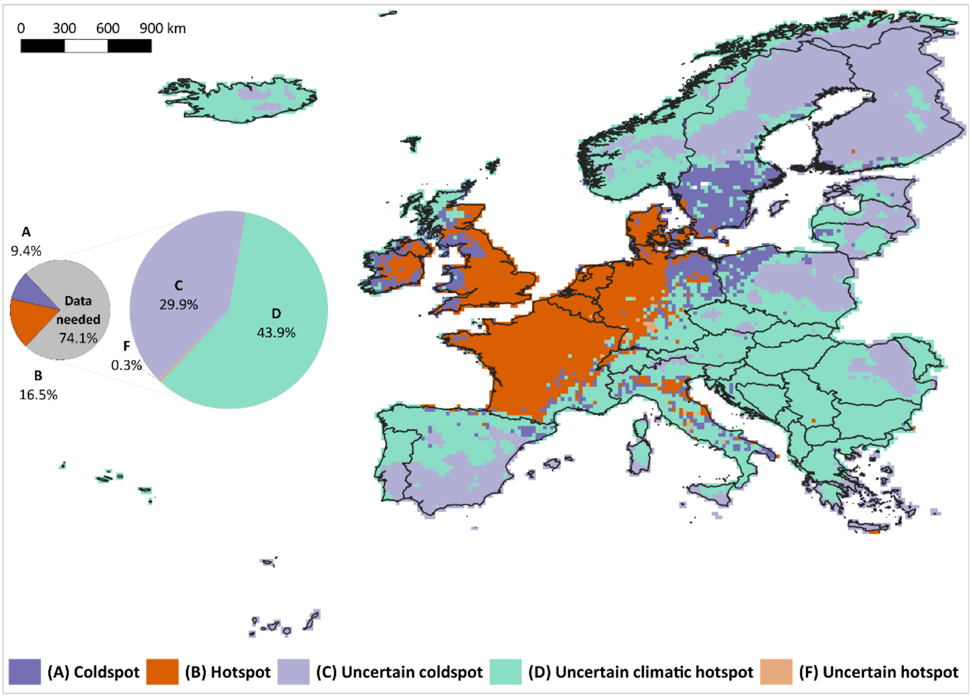

Figure S2.2.7. Priority management areas obtained from the application of the classification criteria described in Fig.1 (main text). The pie-chart to the left represents the proportion of grid-cells that belong to each class after aggregating all the categories under the 'data-needed' group, i.e. zones C to F. The pie-chart to the right represents the percentage of grid-cells within each zone in the 'data-needed' group, i.e. zones C to F (grey area). No grid-cell was classified as E (uncertain environmental hotspot) according to our criteria. *Certain+NA* datasets were used to fit global and European models. This figure was generated with QGIS v.3.2.3[7] (www.qgis.org).

SI 3: Sources of uncertainty

SI 3.1. Uncertainty associated with environmental predictors

Methods

To accurately fit and predict an SDM we need to make sure the models are extrapolated to areas where environmental conditions are similar to those where the model was fitted. To evaluate how different the environmental conditions are outside our sets of presence points, we calculated the multivariate environmental similarity surface (MESS; Elith et al. 2010) using the 'mess' function within the *dismo* R-package[10]. This calculation shows how similar each pixel is in relation to a reference set of points (presence points for each species, in our case) for the values of a set of predictor variables. Pixels with negative values indicate that at least one variable has a value outside the range of environments of the reference set, suggesting these are new environments. Pixels within the environmental range of a species, but in relatively unusual environments, will have a smaller similarity value than those in environments that are more common. Even in areas where there are not presence points (i.e. not considered as reference points for this analysis), pseudo-absences were selected to fit SDMs. Therefore, the set of environmental conditions used to fit the models was actually broader than those shown here; thus, the extrapolations of SDM predictions were made within these areas. We carried out this analysis considering either *certain* or *certain+NA* datasets within Europe as the reference set of points.

Results

Certain dataset

Our results showed that considering all the species together, some areas of eastern Europe, north of Scandinavia, Iceland and the Iberian Peninsula presented the most different environmental conditions (Fig. S3.1.1b). *Neovison vison* and *Branta canadensis* showed overall unusual environments, homogeneously distributed, but with only small areas that presented dissimilar values. *Myocastor coypus, Psittacula krameri* and *Trachemys scripta* exhibited large areas of similar environments all over Europe (Fig. S3.1.1a).

Certain+NA dataset

The general patterns of the distribution of dissimilar environments were similar to those considering the *certain* dataset, with areas of eastern Europe, north of Scandinavia, Iceland and the Iberian Peninsula exhibiting the most different environmental conditions when looking at all species together (Fig. S3.1.2b). In this case, the environmental conditions were particularly well covered and were similar to the reference set of presence points for birds, and also for *N.vison* and *Ondatra zibethicus,* which showed nearly no dissimilarities (Fig. S3.1.2a). Dissimilar areas within the Iberian Peninsula and Scandinavia for *Sciurus carolinensis* and *Lithobates catesbeianus* remained using this dataset.

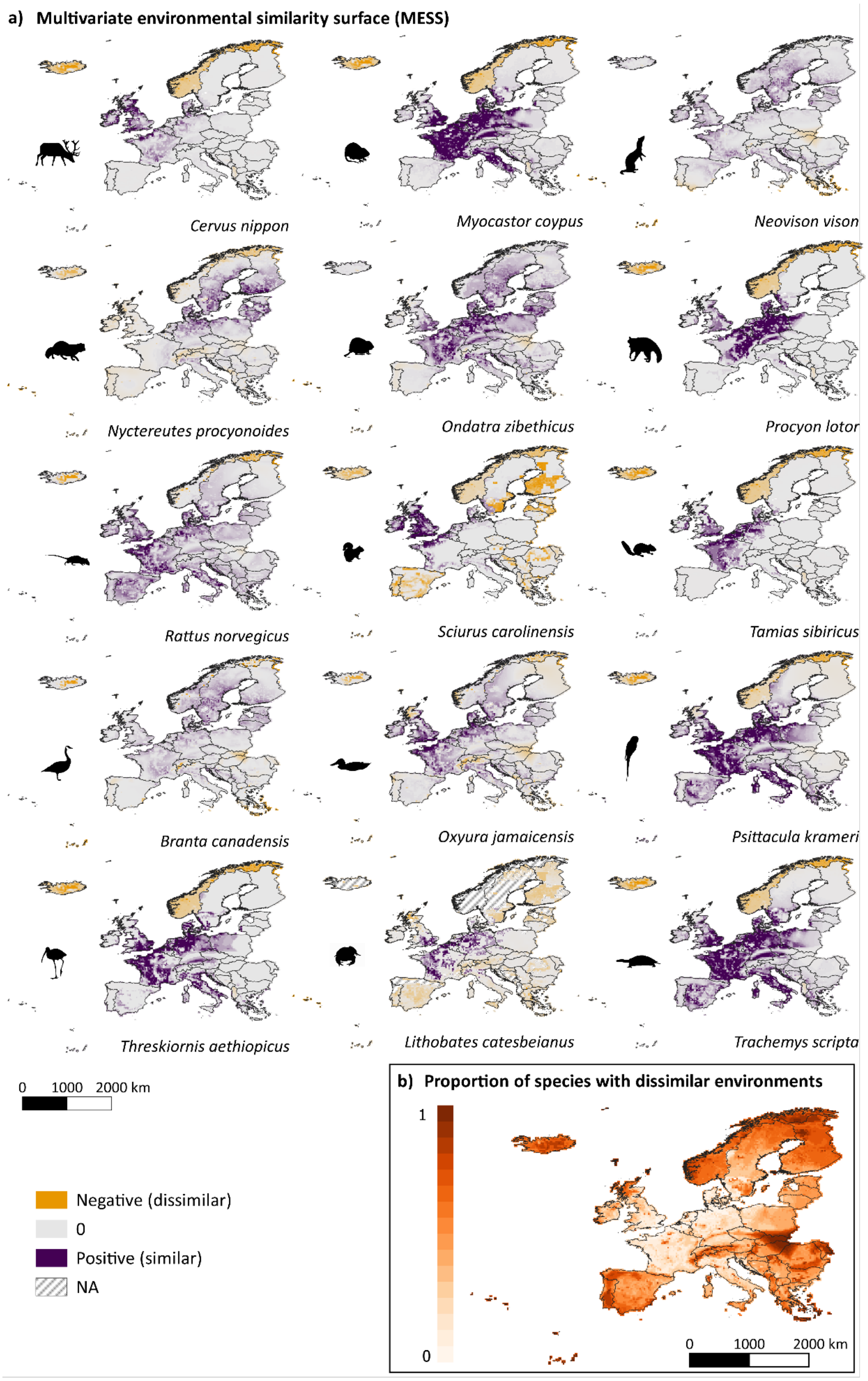

Figure S3.1.1. Multivariate environmental similarity surfaces calculated from the *certain* datasets for each species (a). Orange pixels indicate dissimilar areas. Purple pixels show similar areas. Grey pixels cannot be defined in either of the previous categories, they represent marginal environments. Panel (b) shows the proportion between the count of negative values per pixel and the total number of IATV reported in each grid-cell. This figure was generated with QGIS v.3.2.3[7] (www.qgis.org).

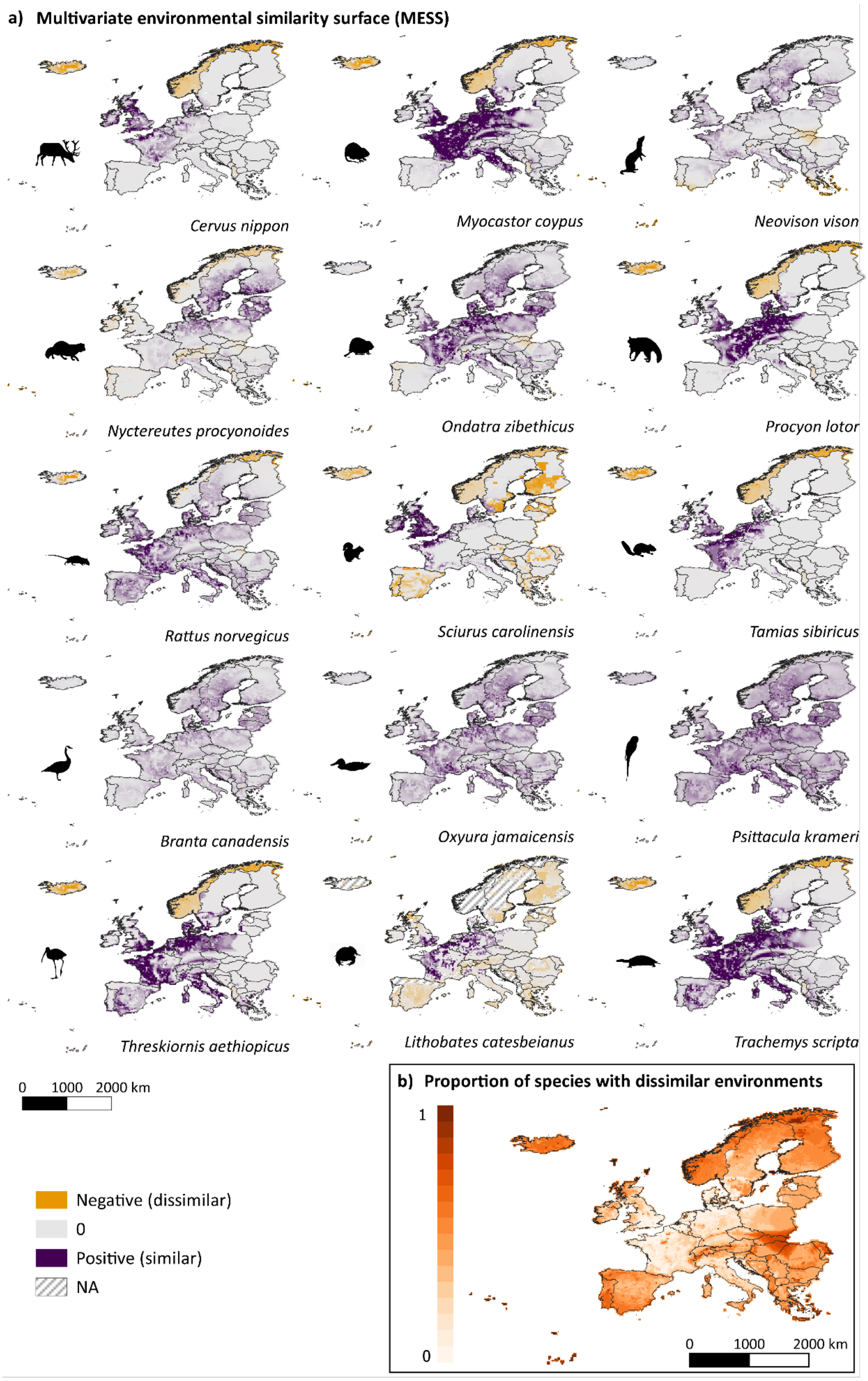

Figure S3.1.2. Multivariate environmental similarity surface calculated from *certain+NA* datasets for each species (a). Orange pixels indicate dissimilar areas. Purple pixels show similar areas. Grey pixels cannot be defined in either of the previous categories, they represent marginal environments. Panel (b) shows the proportion between the count of negative values per pixel and the total number of IATV reported in each grid-cell. This figure was generated with QGIS v.3.2.3[7] (www.qgis.org).

SI 3.2. Uncertainty associated with occurrence data of species

SI 3.2.1 GBIF geographic bias

Methods

Open-access databases such as GBIF are built upon citizen-science data, opportunistic surveys, and other non-systematic sampling. Therefore, some uncertainly is expected to be caused by taxonomic and geographic bias in data collection, and a common lack of reports on species true absences[11–13]. However, GBIF offers significant and valuable advantages, since it covers large geographic regions and taxa with comparable information on species occurrence. In our study, we selected only data coming from direct or machine observations and compatible with our spatial resolution. Moreover, we only held one record per grid-cell to correct for unknown survey efforts. Then, we applied an SDM method that accounted for non-recorded absences, particularly designed for invasive species[14]. Moreover, we included accessibility as a predictor at the European level because this is a known factor positively related to the presence of invasive species[15].

One of the principal issues with presence-only data is the unknown source of spatial bias that makes it difficult to apply corrections. Some initiatives have been proposed in the literature, but their implementation for invasive species remains unclear[16,17]. Records of invasive and native species can present differences in their spatial distribution. On the one hand, sampling protocols may be different due to the diverse objectives behind the monitoring of native vs. native species. On the other hand, opportunistic observations tend to be biased towards rare species; thus, invasive species could be over-represented in this kind of data. Consequently, assuming equal sampling spatial patterns in both cases could be misleading. To illustrate this issue and to better understand the bias of the occurrence data used in this research, we calculated an ignorance score using taxonomic reference groups for each of our target species (Eq. S1; Ruete 2015). We used the taxonomic level of family as the reference group for each species. We downloaded direct or machine observations within our study area in Europe for each family from GBIF (Table S3.2.1.1; www.gbif.org). Because *Myocastor coypus* and *Procyon lotor* are the only species of their family in Europe, we discarded them from the analyses. We also computed the same ignorance scores for each individual species to compare between the ignorance distribution of each species and that of its family.

Namely, we applied the half-ignorance algorithm[17] to estimate the ignorance score per pixel by making data relative to a reference number of observations considered to suffice a score reduction by half, defined as:

$$I_i = \frac{O_{0.5}}{(N_i + O_{0.5})} \qquad \textit{Eq. S1}$$

where I_i is the ignorance score, $O_{0.5}$ is a reference number of observations that is considered to be enough to reduce the ignorance score by half, and N_i is the raw count of occurrences from each family per grid-cell.

We set $O_{0.5}$ = 1, meaning that the number of observations needed to consider the absence of reports of a target species in any grid-cell is 50% due to a true absence from the site, and 50% due to a failure to detect the species.

Results

Results showed a trend of better-sampled areas towards Western Europe and the British Islands, when looking at all the families together (Fig. S3.2.1.1b). Birds were the most homogeneously sampled group, often following a pattern characteristic of data retrieved from atlas (*Psittacidae* and *Threskiornidae*; Fig. S3.2.1.1a). *Anatidae* were highly reported all over Europe but still with some remarkable gaps in eastern Europe and inland Iceland. All the mammalian families showed similar sampling patterns, with *Sciuridae* underreported or undersampled in the Iberian Peninsula . Data from amphibians of the family *Ranidae* were scarce in Iceland and western Europe. Data of the reptilian family *Emydidae* were mainly concentrated in Western Europe (Fig. S3.2.1.1.a).

For native species, the dark blue areas in Fig. S3.2.1.1 (low ignorance values) would indicate where a family is generally well-reported and thus, we should expect the same for all of the species within that family. After combining this information with that on Fig. S3.2.1.2 or Fig. S3.2.1.3, the overlap of a beige/yellow area (high ignorance value) and the aforementioned blue area would suggest the ignorance is more likely to be a real absence in that area than a lack of reports for this particular species. Alternatively, regions presenting high ignorance values in Fig. S3.2.1.1, and Fig. S3.2.1.2 or Fig. S3.2.1.3 (depending on whether we are working with *certain* or *certain+NA* datasets) are generally undersampled and no information is obtained from them. In general, what we defined as *uncertain coldspots* and *uncertain climatic hotspots* coincided generally with areas of a high ignorance (Figs. 2 and S3.2.1.1, Table 4).

Table S3.2.1.1. Species of study in this research, the family they belong to, and the number of species within each family represented in Europe according to GBIF (www.gbif.org).

Species	Family	No. species in Europe
**Mammals**		
Cervus nippon	*Cervidae*	13
Mustela vison	*Mustelidae*	19
Rattus norvegicus	*Muridae*	24
Myocastor coypus	*Myocastoridae*	1
Nyctereutes procyonoides	*Canidae*	10
Ondatra zibethicus	*Cricetidae*	28
Procyon lotor	*Procyonidae*	1
Sciurus carolinensis	*Sciuridae*	26
Tamias sibiricus	*Sciuridae*	26
**Birds**		
Branta canadensis	*Anatidae*	153
Oxyura jamaicensis	*Anatidae*	153
Psittacula krameri	*Psittacidae*	64
Threskiornis aethiopicus	*Threskiornithidae*	14
**Amphibians**		
Lithobates catesbeianus	*Ranidae*	26
**Reptiles**		
Trachemys scripta	*Emydidae*	15

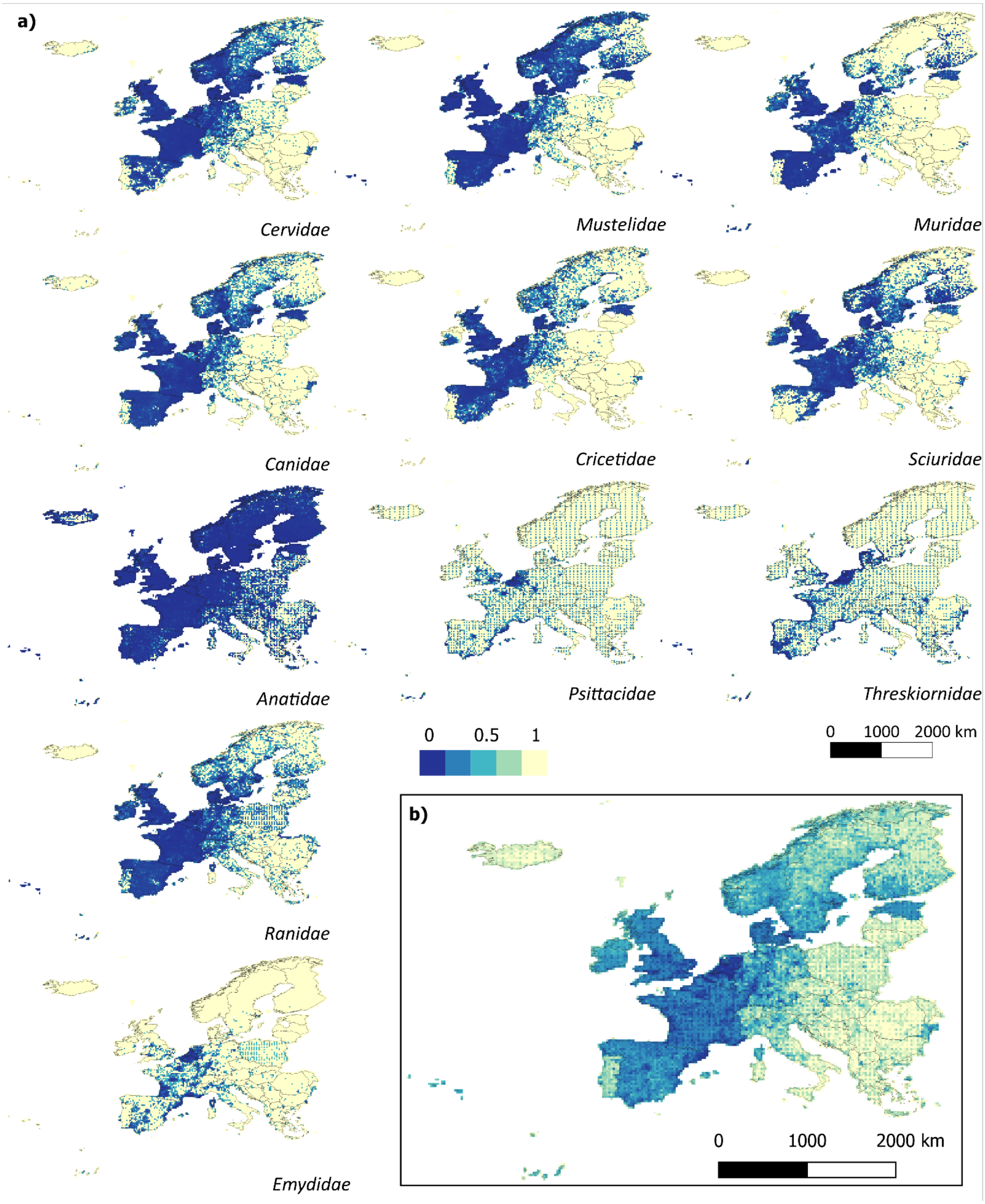

Figure S3.2.1.1. Ignorance maps representing the half-ignorance score per grid cell for the families of the 15 IATV (invasive alien terrestrial vertebrates) species studied (a), and the average value for all families (b). Dark blue pixels indicate a low level of ignorance (min.=0), beige/yellow pixels indicate a high level of ignorance (max.=1). This figure was generated with QGIS v.3.2.3[7] (www.qgis.org).

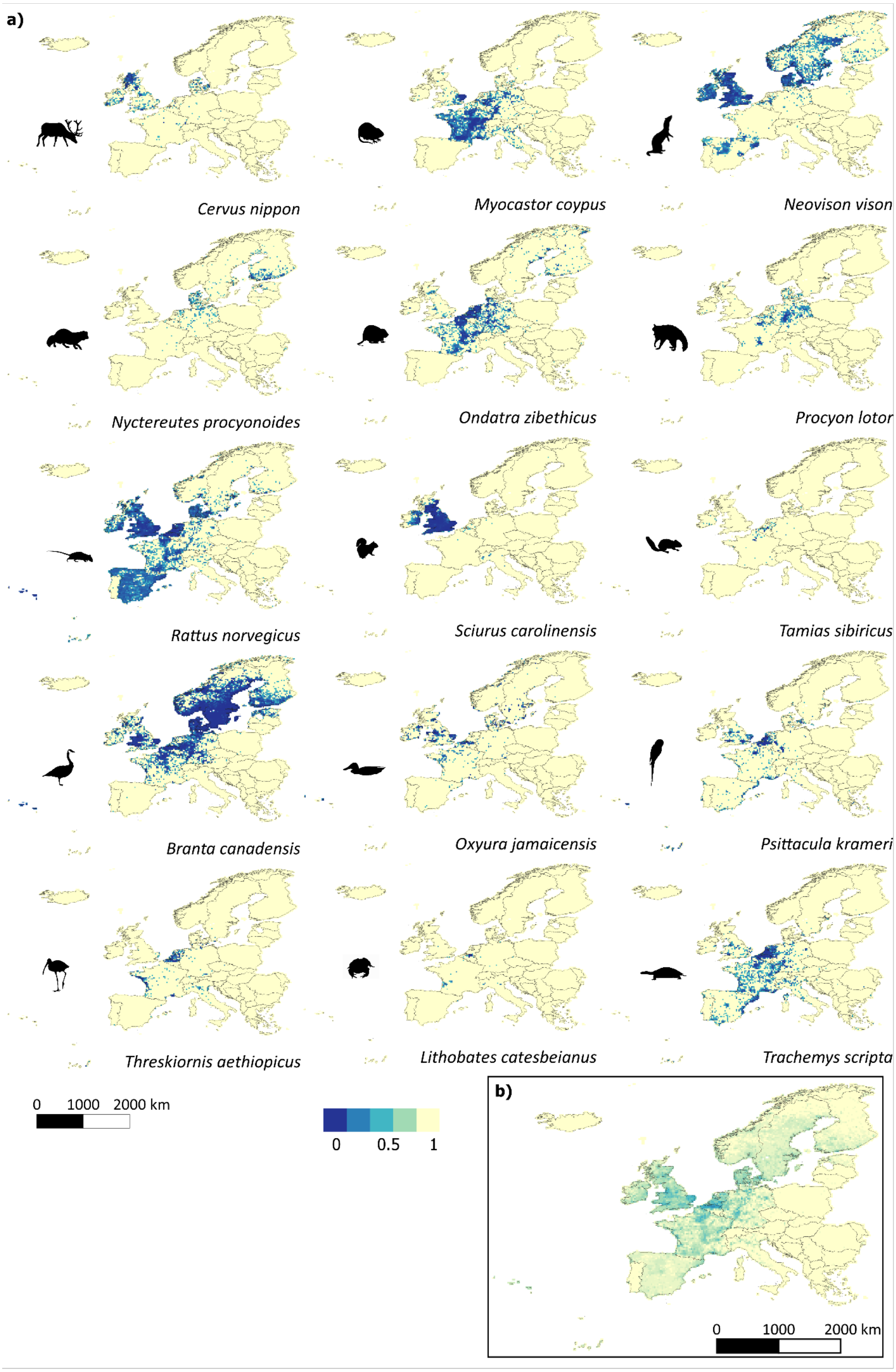

Figure S3.2.1.2. Ignorance maps representing the half-ignorance score per grid cell for 15 IATV (invasive alien terrestrial vertebrates; a) and the average value for all families (b). Dark blue pixels indicate a low level of ignorance (min.=0), beige/yellow pixels indicate a high level of ignorance (max.=1). *Certain* datasets were used. This figure was generated with QGIS v.3.2.3[7] (www.qgis.org).

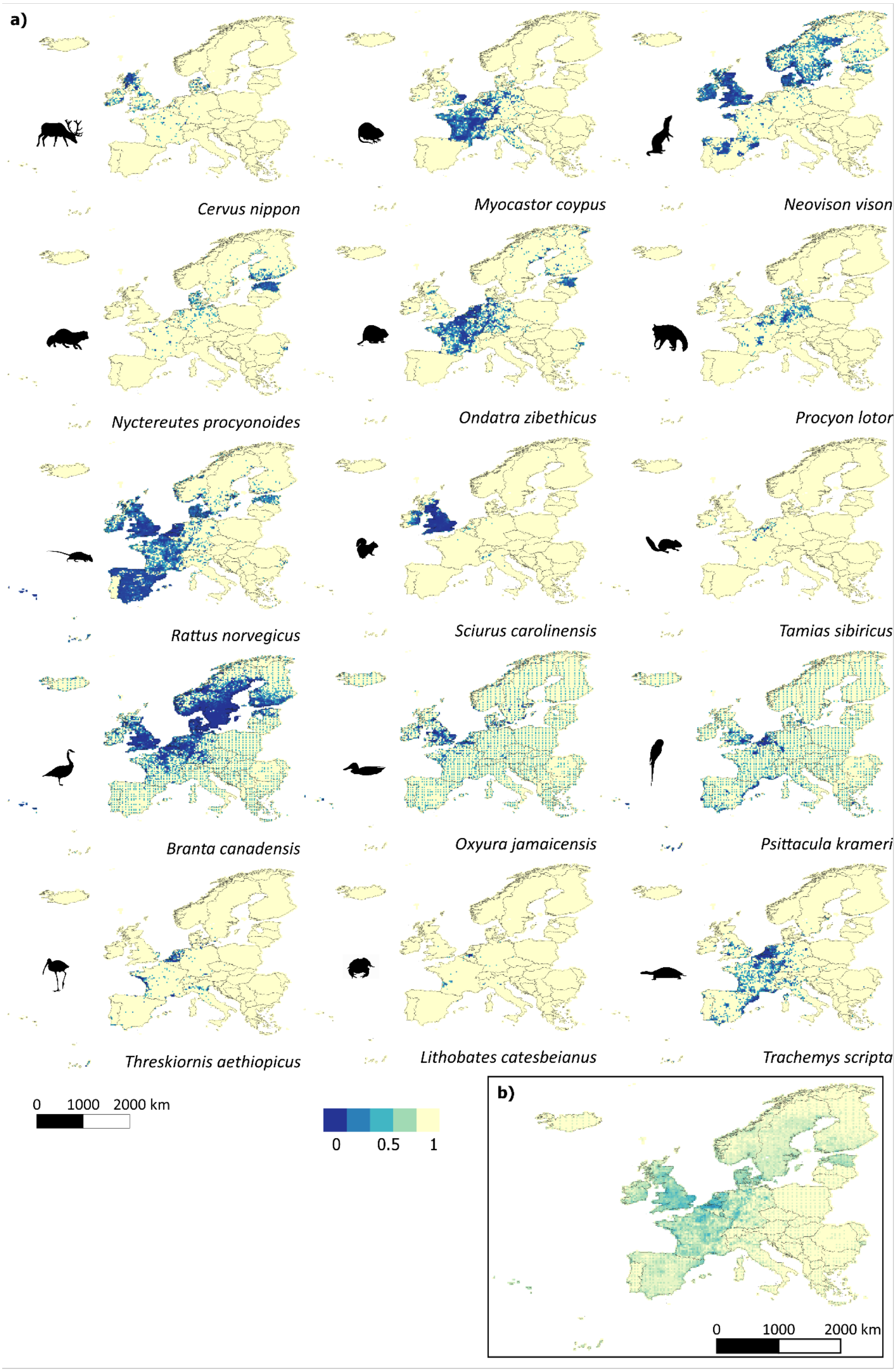

Figure S3.2.1.3. Ignorance maps representing the half-ignorance score per grid cell for 15 IATV (invasive alien terrestrial vertebrates; a) and the average value for all families (b). Dark blue pixels indicate a low level of ignorance (min.=0), beige/yellow pixels indicate a high level of ignorance (max.=1). *Certain+NA* datasets were used. This figure was generated with QGIS v.3.2.3[7] (www.qgis.org).

SI 3.2.2 Additional data sources

To add further insights on the agreement between different sources of data, we conducted a rough comparison between the Global Biodiversity Information Facility (GBIF) data used in this research and the Invasive Species Compendium developed by the Centre for Agriculture and Bioscience International (CABI; https://www.cabi.org/isc/). We compiled the number of countries where each species of IATV was reported in both data sources either as native or invasive. In this comparison, we obtained a low agreement at the global level between our GBIF datasets and CABI (Table S3.2.2.1), with values ranging from 0.54 (*certain* dataset) to 0.64 (*certain* + NA dataset). Conversely, the agreement was higher for the European data, with values between 0.71 (*certain* dataset) and 0.78 (*certain* + NA dataset). However, although these two sources of information might be seen as complementary, CABI does not include georeferenced points of occurrences. Therefore, the countries included in this dataset may refer to few or numerous observations, which makes unfeasible to know the amount of information missed from excluding those countries included in CABI but not in GBIF.

Table S3.2.2.1 Number of countries where each IATV species is reported, according to GBIF and CABI. Global refers to the total of countries in the world, including native and invasive areas. Europe refers to the countries included in our study area. *Cert* shows the number of countries included when using the *certain* dataset. *Cert+NA* shows the number of countries included when using the *certain+NA* dataset. Overlap indicates the proportion of countries included in GBIF respect to the included in CABI.

	**Global**					**Europe**				
Species	Number of countries (N)			Overlap (N_{GBIF}/N_{CABI})		Number of countries (N)			Overlap (N_{GBIF}/N_{CABI})	
	**GBIF**		**CABI**			**GBIF**		**CABI**		
	cert	*cert+NA*		*cert*	*cert+NA*	*cert*	*cert+NA*		*cert*	*cert+NA*
**Mammals**										
Cervus nippon	12	15	20	0.50	0.55	9	10	10	0.70	0.80
Myocastor coypus	32	33	45	0.58	0.58	15	16	16	0.69	0.69
Neovison vison	23	24	40	0.55	0.58	18	19	30	0.57	0.60
Nyctereutes procyonoides	18	20	36	0.42	0.47	13	14	23	0.43	0.48
Ondatra zibethicus	27	31	34	0.65	0.74	21	24	21	0.81	0.90
Procyon lotor	25	25	35	0.56	0.56	11	11	15	0.47	0.47
Rattus norvegicus	54	57	75	0.32	0.33	24	26	10	0.80	0.90
Sciurus carolinensis	14	14	6	1.00	1.00	8	8	3	1.00	1.00
Tamias sibiricus	10	11	14	0.46	0.54	7	7	6	0.67	0.67
**Birds**										
Branta canadensis	30	56	50	0.55	0.78	21	36	25	0.80	0.96
Oxyura jamaicensis	24	66	46	0.51	0.80	12	35	18	0.61	1.00
Psittacula krameri	50	105	78	0.57	0.88	15	34	11	0.91	1.00
Threskiornis aethiopicus	32	58	53	0.44	0.81	12	12	8	0.88	0.88
**Amphibians**										
Lithobates catesbeianus	21	22	48	0.44	0.46	7	7	10	0.70	0.70
**Reptiles**										
Trachemys scripta	50	56	59	0.56	0.58	20	21	16	0.69	0.69
Average				**0.54**	**0.64**				**0.71**	**0.78**

SI 3.3 Uncertainty associated with variability of predictions – CV of ensemble predictions

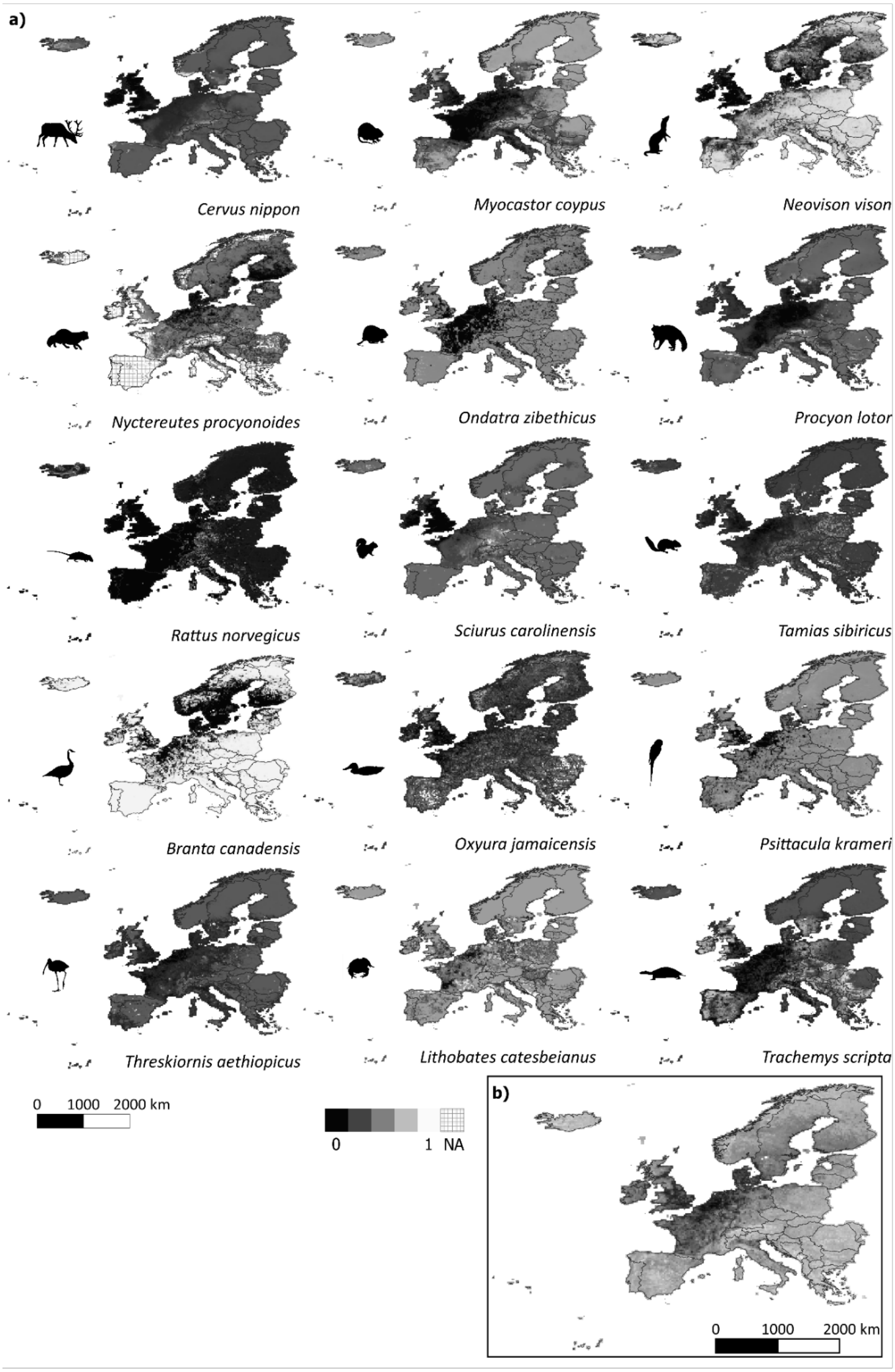

Figure S3.3.1. Coefficients of variation of the environmental suitability predictions from the European ensemble SDMs for each IATV (invasive alien terrestrial vertebrates); a) and their average (b). Models were fitted with the *certain+NA* dataset for the global model, and with the *certain* dataset for the European model. Values were normalized between 0 and 1. This figure was generated with QGIS v.3.2.3[7] (www.qgis.org).

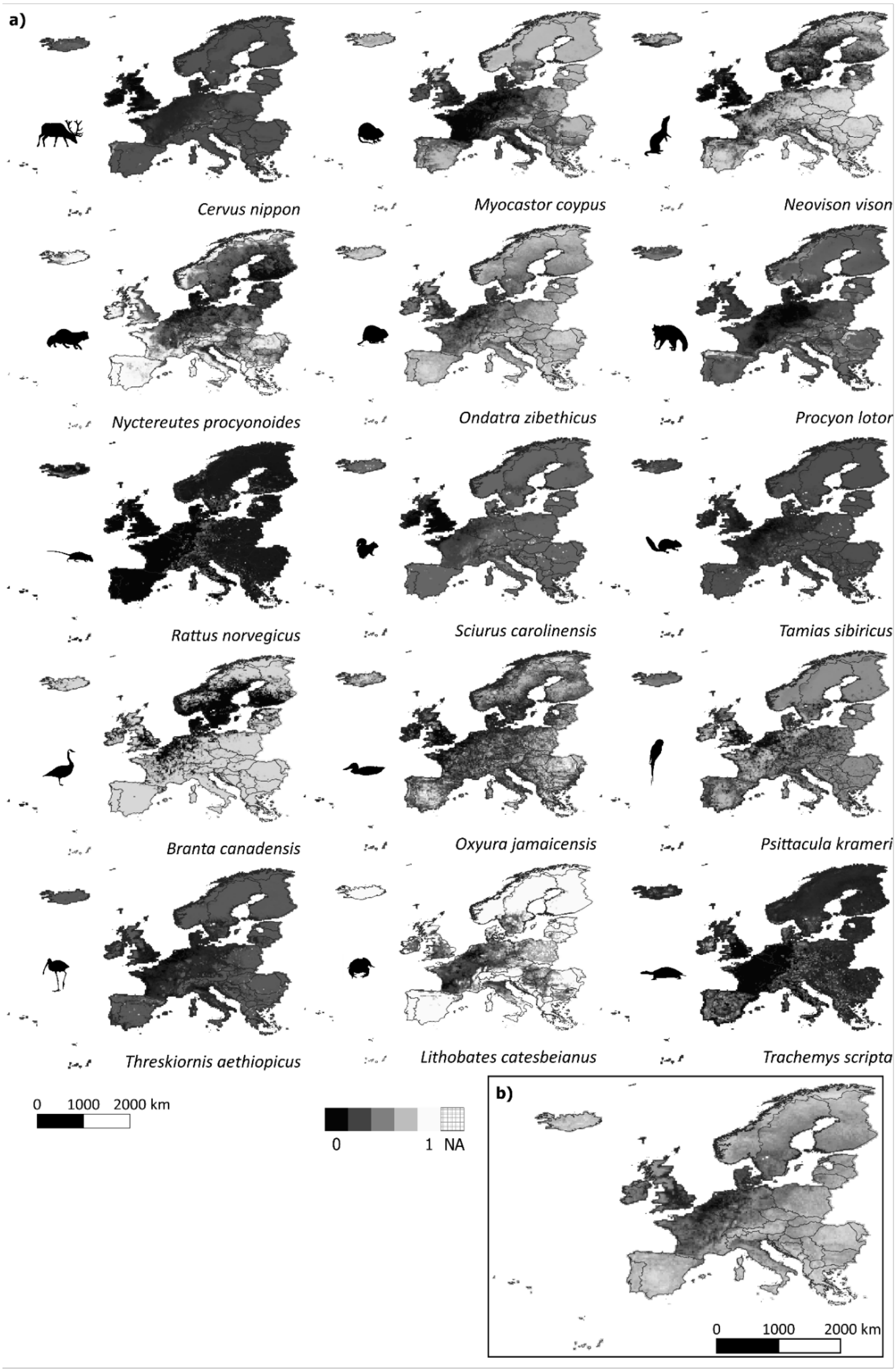

Figure S3.3.2. Coefficients of variation of the environmental suitability predictions from the European ensemble SDMs for each IATV (invasive alien terrestrial vertebrates; a) and their average (b). Models were fitted with the *certain* datasets for the global and European models. Values were normalized between 0 and 1. This figure was generated with QGIS v.3.2.3[7] (www.qgis.org).

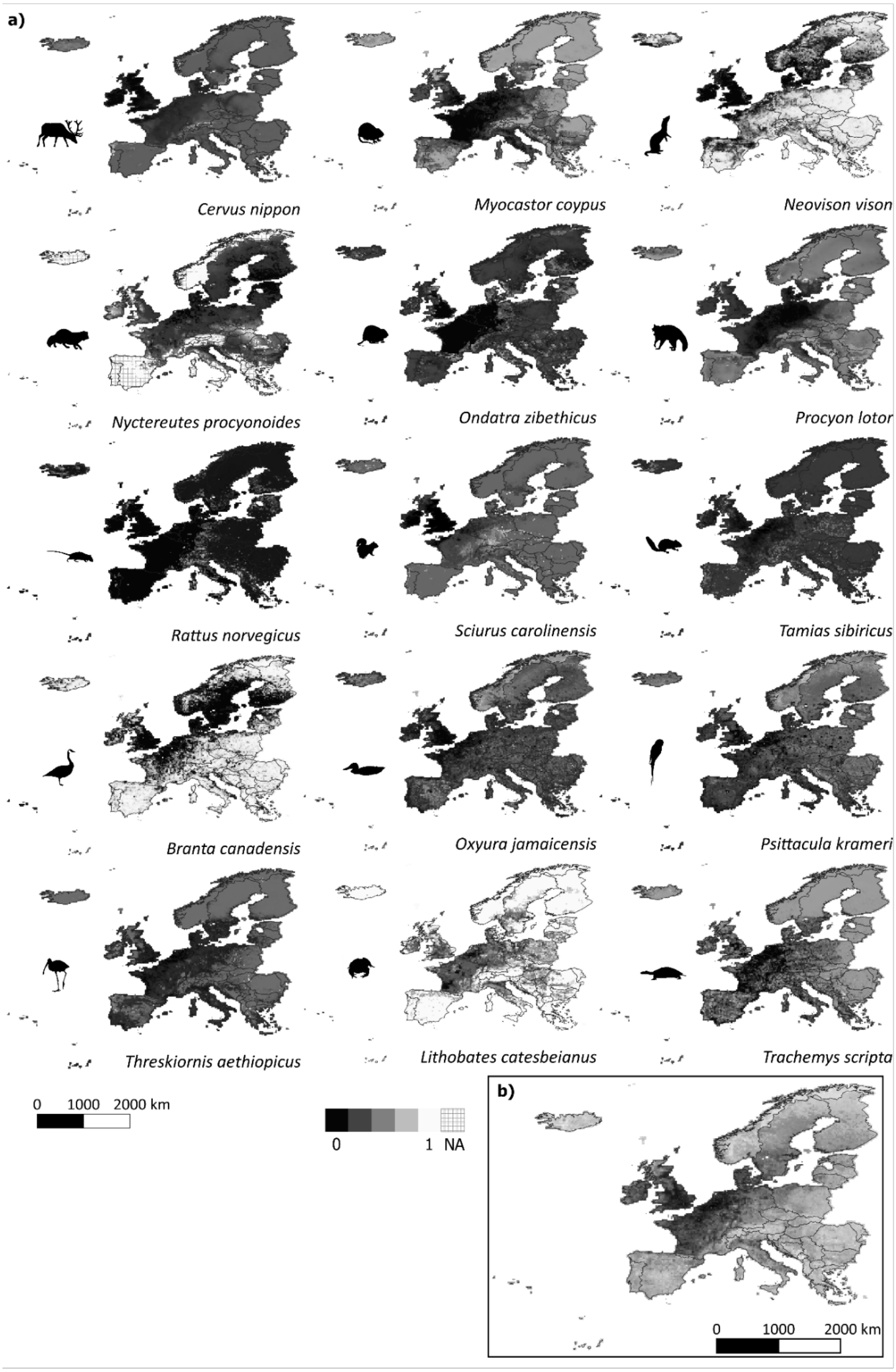

Figure S3.3.3. Coefficients of variation of the environmental suitability predictions from the European ensemble SDMs for each IATV (invasive alien terrestrial vertebrates; a) and their average (b). Models were fitted with the *certain+NA* datasets for the global and European models. Values were normalized between 0 and 1. This figure was generated with QGIS v.3.2.3[7] (www.qgis.org).

References

1. Karger, D. N. *et al.* Climatologies at high resolution for the earth's land surface areas. *Sci. Data* **4**, 1–20 (2017).

2. Hurtt, G. C. *et al.* Harmonization of land-use scenarios for the period 1500 – 2100 : 600 years of global gridded annual land-use transitions , wood harvest , and resulting secondary lands. *Clim. Change* **109**, 117–161 (2011).

3. Natural Earth. Rivers and lake centerlines. (2018). Available at: https://www.naturalearthdata.com/downloads/10m-physical-vectors/10m-rivers-lake-centerlines/.

4. EEA. Corine Land Cover (CLC), Copernicus Land Monitoring Service. (2018).

5. LP DAAC. Global 30 arc-second elevation data set GTOPO30. *Land Process Distributed Active Archive Center* (2004). Available at: http://edcdaac.usgs.gov/gtopo30/gtopo30.asp. (Accessed: 1st September 2017)

6. Nelson, A. Estimated travel time to the nearest city of 50,000 or more people in year 2000. *Global Environment Monitoring Unit-Joint Research Centre of the European Comission* (2008). Available at: http://bioval.jrc.ec.europa.eu/products/gam/. (Accessed: 1st October 2017)

7. QGIS Development Team. QGIS Geographic Information System. (2018).

8. Huang, Q., Fleming, C. H., Robb, B., Lothspeich, A. & Songer, M. Ecological Informatics How different are species distribution model predictions ?— Application of a new measure of dissimilarity and level of significance to giant panda Ailuropoda melanoleuca. *Ecol. Inform.* **46**, 114–124 (2018).

9. Elith, J., Kearney, M. & Phillips, S. The art of modelling range-shifting species. *Methods Ecol. Evol.* **1**, 330–342 (2010).

10. Hijmans, R. J., Phillips, S., Leathwick, J. R. & Elith, J. Dismo package for R, version 1.1-4. (2017). doi:10.1016/j.jhydrol.2011.07.022.

11. Amano, T., Lamming, J. D. L. & Sutherland, W. J. Spatial Gaps in Global Biodiversity Information and the Role of Citizen Science. *Bioscience* **66**, 393–400 (2016).

12. Troudet, J., Grandcolas, P., Blin, A., Vignes-Lebbe, R. & Legendre, F. Taxonomic bias in biodiversity data and societal preferences. *Sci. Rep.* **7**, 1–14 (2017).

13. Rocchini, D. *et al.* Accounting for uncertainty when mapping species distributions: The need for maps of ignorance. *Prog. Phys. Geogr.* **35**, 211–226 (2011).

14. Gallien, L., Douzet, R., Pratte, S., Zimmermann, N. E. & Thuiller, W. Invasive species distribution models – how violating the equilibrium assumption can create new insights. *Glob. Ecol. Biogeogr.* **21**, 1126–1136 (2012).

15. Seebens, H. *et al.* Global rise in emerging alien species results from increased accessibility of new source pools. *Proc. Natl. Acad. Sci. U. S. A.* **115**, E2264–E2273 (2018).

16. Stolar, J. & Nielsen, S. E. Accounting for spatially biased sampling effort in presence-only species distribution modelling. *Divers. Distrib.* **21**, 595–608 (2015).

17. Ruete, A. Displaying bias in sampling effort of data accessed from biodiversity databases using ignorance maps. *Biodivers. Data J.* **3**, (2015).
